# Silencing of miR-138-5p sensitizes bone anabolic action to mechanical stimuli

**DOI:** 10.7150/thno.53009

**Published:** 2020-10-30

**Authors:** Zhihao Chen, Fan Zhao, Chao Liang, Lifang Hu, Dijie Li, Yan Zhang, Chong Yin, Lei Chen, Luyao Wang, Xiao Lin, Peihong Su, Jianhua Ma, Chaofei Yang, Ye Tian, Wenjuan Zhang, Yu Li, Songlin Peng, Weiyi Chen, Ge Zhang, Airong Qian

**Affiliations:** 1 Lab for Bone Metabolism, Xi'an Key Laboratory of Special Medicine and Health Engineering, Key Lab for Space Biosciences and Biotechnology, Research Center for Special Medicine and Health Systems Engineering, NPU-UAB Joint Laboratory for Bone Metabolism, School of Life Sciences, Northwestern Polytechnical University, Xi'an, Shaanxi, 710072, China.; 2 Northwestern Polytechnical University - Hong Kong Baptist University Joint Research Centre for Translational Medicine on Musculoskeletal Health in Space, Xi'an, Shaanxi 710072, China.; 3 Law Sau Fai Institute for Advancing Translational Medicine in Bone and Joint Diseases, School of Chinese Medicine, Hong Kong Baptist University, Hong Kong, SAR, 999077, China.; 4 Department of Spine Surgery, Shenzhen People's Hospital, Southern University of Science and Technology, Jinan University School of Medicine, Shenzhen, 518020, China.; 5 Shanxi Key Laboratory of Material Strength & Structural Impact, College of Biomedical Engineering, Taiyuan University of Technology, Taiyuan, 030024, China.; 6 Department of Biology, Southern University of Science and Technology, Shenzhen, 518055, China.

**Keywords:** mechanoresponsive, miR-138-5p, sensitize, bone formation, osteoporosis

## Abstract

Emerging evidence is revealing that microRNAs (miRNAs) play essential roles in mechanosensing for regulating osteogenesis. However, no mechanoresponsive miRNAs have been identified in human bone specimens.

**Methods:** Bedridden and aged patients, hindlimb unloaded and aged mice, and Random Positioning Machine and primary aged osteoblasts were adopted to simulate mechanical unloading conditions at the human, animal and cellular levels, respectively. Treadmill exercise and Flexcell cyclic mechanical stretching were used to simulate mechanical loading* in vivo* and *in vitro*, respectively.

**Results:** Here, we found increased miR-138-5p levels with a lower degree of bone formation in bone specimens from bedridden and aged patients. Loss- and gain-of-function studies showed that miR-138-5p directly targeted microtubule actin crosslinking factor 1 (MACF1) to inhibit osteoblast differentiation under different mechanical conditions. Regarding translational medicine, bone-targeted inhibition of miR-138-5p attenuated the decrease in the mechanical bone anabolic response in hindlimb unloaded mice. Moreover, bone-targeted inhibition of miR-138-5p sensitized the bone anabolic response to mechanical loading in both miR-138-5p transgenic mice and aged mice to promote bone formation.

**Conclusion:** These data suggest that miR-138-5p as a mechanoresponsive miRNA accounts for the mechanosensitivity of the bone anabolic response and that inhibition of miR-138-5p in osteoblasts may be a novel bone anabolic sensitization strategy for ameliorating disuse or senile osteoporosis.

## Introduction

Mechanical stimuli are essential for maintaining bone metabolism and play key roles in regulating osteogenic differentiation and bone formation 1-3. In clinical bedridden and aged patients who lack mechanical stimuli, bone mineral density (BMD) has been recorded over time to decrease persistently 4, 5. Mechanical stimuli sensed by osteoblasts can be transduced into intracellular biochemical signals to regulate gene expression and cell differentiation 2. MicroRNAs (miRNAs) are ~22 nt non-protein coding RNAs that regulate gene expression to coordinate a broad spectrum of biological processes 6-8. Increasing evidence demonstrates that several mechanosensitive miRNAs are involved in osteogenesis by targeting key transcription factors or signaling pathways 9-11. It has been reported that mechanosensitive miR-103a directly targeted the key transcription factor-Runx2 (runt-related transcription factor 2) and mechanosensitive miR-154-5p directly targeted a key signaling pathway-Wnt signaling pathway to inhibit osteogenic differentiation or bone formation 12, 13. However, there are still no mechanosensitive miRNAs identified in clinical bone specimens that have been systematically described in terms of their potential roles in bone anabolic activity under different mechanical conditions. In addition, in consideration of the limited effect exercise has on degenerative osteoporosis, whether these mechanosensitive miRNAs could be targeted to sensitize bone to mechanical stimuli and to further improve the effectiveness of exercise on osteoporosis are also important clinical questions.

In this study, we screened the reported mechanosensitive miRNAs and identified that the differential expression of miR-138-5p is highest in the bone tissue of hindlimb unloaded mice. We further clinically identified that miR-138-5p is a mechanoresponsive miRNA that is negatively correlated with bone formation in bone specimens from bedridden and aged male osteoporotic patients. Further experiments revealed microtubule actin crosslinking factor 1 (MACF1) as a downstream target of miR-138-5p in mechanosensitivity and bone anabolic action regulation. Finally, bone-targeted inhibition of miR-138-5p enhanced the effect of exercise on bone formation and showed that miR-138-5p is a potential therapeutic mechano-sensitized target for the treatment of disuse or senile osteoporosis.

## Materials and methods

### Human bone specimen collection

Human bone specimens were collected from the Shenzhen People's Hospital. Bedridden age-matched (50-59 years old) male patients (n = 6 for each group) and aged osteoporotic (non-bedridden) male patients (n = 6 for each group) were included in our study. Subjects with liver or kidney disease, osteoarthritis, inflammation or systemic inflammatory disease, malignancy, chronic corticosteroid use, or with other severe diseases in the previous five years were excluded from our study (exclusion criteria). All the clinical procedures were approved by the Committees of Clinical Ethics in the Shenzhen People's Hospital. We obtained informed consent from the participants.

### Mice and mouse models

#### Mice

All mice used in this study were male mice purchased from the Laboratory Animal Center of Air Force Medical University (formerly the Fourth Military Medical University, Xi'an, Shaanxi, China). The mice were maintained in SPF conditions and were given free access to water and ^60^Co-irradiated feed. The mice were handled according to the 3Rs principle, and euthanized by pentobarbital sodium injection (1 g/kg body weight) or CO_2_ inhalation. All mouse experiments were performed according to the Guide for the Care and Use of Laboratory Animals and with the permission of the Laboratory Animal Ethics & Welfare Committee of Northwestern Polytechnical University.

#### Hindlimb unloaded (HU)/reloaded mouse model

The hindlimb unloaded mouse model was constructed using 8-week-old male C57BL/6 mice according to previous reports 14. Briefly, a clip-shaped holder was reformed to secure the tail of each mouse, and the mice were then suspended with a chain at a degree of 30° in a custom-made suspension cage. The mice were free to move in the cage and access feed and water. The suspension period lasted for 4 weeks (unloading period). After suspension, the mice were released from suspension free and housed in a normal cage for another 2 weeks (reloading period).

#### Aged mice model

Naturally aged male C57BL/6 mice were used in this study. The condition of the mice was monitored continuously and until the mice were more than 20 months old, and then unhealthy mice were excluded.

#### Treadmill exercise mice model

A running exercise for the mice was performed using a laboratory animal treadmill (Zhenghua ZH-PT, China). Mice were randomly grouped into sedentary or exercise groups randomly. The treadmill exercise lasted for 4 weeks (5 days/week). The exercise intensity was set to 10 m/min for 20 min (5° upslope) in the first week, and was increased gradually to 15 m/min for 35 min in the fourth-week. The control mice were caged under normal conditions and not subjected to treadmill exercise.

#### miR-138-5p transgenic mouse model

An osteoblast-specific miR-138-5p transgenic (TG) mouse model was constructed by the pronuclear microinjection method. Briefly, the miR-138-5p sequence flanked by the miR-30 loop was cloned into a pRP (Exp) vector downstream of the Runx2 promoter (pRP (Exp)-RUNX2>5'miR30-mmu-miR-138-5p-3'miR30). The recombinant plasmid was then sequenced, linearized, and microinjected into a zygote. Six F0 founder mice were obtained and bred to F3 individually, the miR-138-5p transgenic mouse line was established using the best F0 founder. In this study, +/- mice were used as transgenic mice, and -/- mice were used as WT controls.

### RNA extraction and real-time PCR

High quality total RNA from tissue or cell samples were isolated using the Omega miRNA Kit or Omega Total RNA Kit (Omega, USA). For isolation of total RNA from tissue, samples were pulverized using a mortar and pestle in liquid nitrogen. RNA quality was then determined by ultraviolet spectrophotometry. cDNA was synthesized from 0.5 μg of total RNA using a cDNA synthesis kit (PrimeScript™ RT Reagent Kit, TaKaRa, Japan) following the manufacturer's instructions. Real-time quantitative PCR was performed using the SYBR® Premix Ex Taq™ II kit (TaKaRa, Japan). Relative fold changes of the genes and miRNAs were analyzed by the 2^-ΔΔCT^ method. *Gapdh* and U6 were used as internal controls for the mRNA and miRNA analyses, respectively.

### Micro-computed tomography

**Figure 1:**
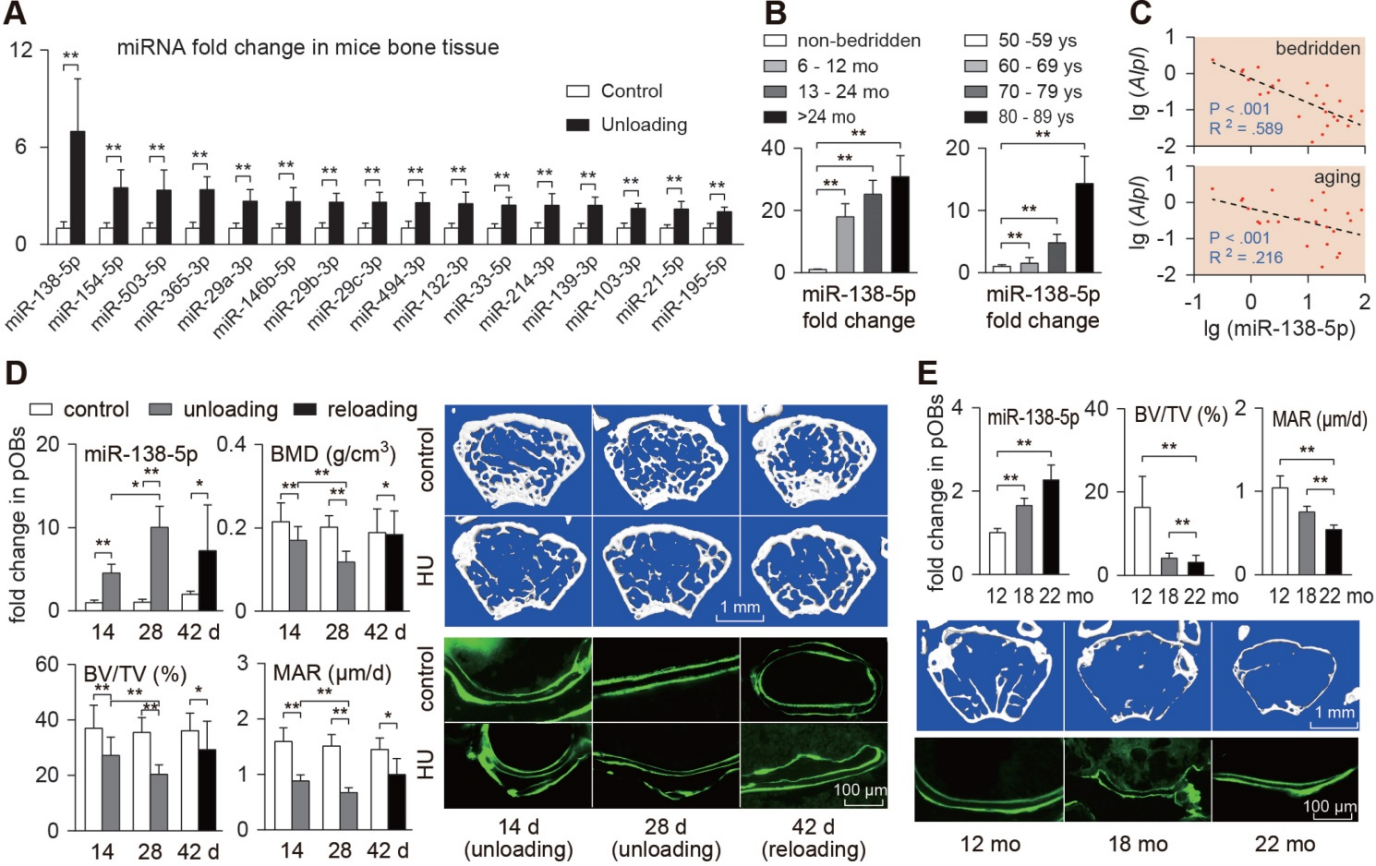
** miR-138-5p is a mechanoresponsive miRNA and negatively correlates with bone formation.** (**A**) Real-time PCR analysis of candidate mechanosensitive miRNAs levels in bone tissues of hindlimb unloaded mice. n = 6 in each group. (**B**) Real-time PCR analysis of miR-138-5p level in bone specimens from bedridden men patients (50-59 years old) with different periods of immobilization (left) or aged men fracture patients (non-bedridden) with different age (right). n = 6 in each group. (**C**) Linear regression analysis of miR-138-5p and *Alpl* association in bone specimens from bedridden (upper) or aged (lower) patients. (**D**) Real-time PCR analysis of miR-138-5p level in primary osteoblasts isolated from hindlimb unloaded and reloaded mice, respectively; Representative images showing trabecular microarchitecture, and microCT statistical analysis of BMD, BV/TV in distal femur of hindlimb unloaded or reloaded mice. Scale bar: 1 mm; Calcein double-labeling and dynamic histomorphometric analysis of MAR in distal femur of hindlimb unloaded and reloaded mice, respectively. Scale bar, 100 µm. n ≥ 6 for each group. BMD, bone mineral density; BV/TV, bone volume fraction; MAR, mineral apposition rate. (**E**) Real-time PCR analysis of miR-138-5p level in primary osteoblasts from 12, 18 and 22-month-old male C57BL/6 mice, respectively. n = 3 for each group; Representative images showing trabecular microarchitecture, and microCT statistical analysis of BV/TV in distal femur of 12, 18 and 22-month-old male C57BL/6 mice. Scale bar: 1 mm; Calcein double-labeling and dynamic histomorphometric analysis of MAR in distal femur of 12, 18 and 22-month-old male C57BL/6 mice, respectively. n ≥ 4 for each group. Scale bar, 100 µm. U6 small nuclear RNA was used as the internal control for miRNAs. For human experiments, Data are represented as mean ± sem. For mice and cells experiments, Data are represented as mean ± s.d. Significances were determined using student's *t*-test between two groups. *P* value less than 0.05 was considered significant in all cases (*P < 0.05, **P < 0.01).

To visualize the bone microarchitecture, which is shown in Figures 1 and 4, fixed femur samples were scanned by a microCT device (General Electric, WI). Briefly, femur tissues were isolated and fully fixed in 4% PFA. The fixed samples were then secured to the sample holder and scanned by the microCT device at an isotropical resolution of 8 μm (Energy: 80 kVp/80 μA; angle of Increment: 0.5°; exposure Time: 3000 ms/frame; and scanning Time: 120 min). Reconstructed data were acquired using built-in software. The region of interest was selected 1 mm above the distal growth plate. Mineral density (bone mineral density, BMD; and bone volume to tissue volume, BV/TV) and trabecular microarchitecture parameters (trabecular number, Tb.N; trabecular thickness, Tb.Th; and trabecular spacing, Tb.Sp) were analyzed using the MicroView software.

To visualize bone microarchitecture, which is shown in Figures 5 and 6 and Figure S4, fixed femur samples were scanned by a microCT device (eXplore Locus SP, GE; μCt40, Scanco, Switzerland) at a voxel size of 10 μm. The scanning protocol was performed according to previous reports 15, 16 as follows: 423 slices were scanned at the region of the distal femur beginning at the growth plate and extending proximally along the femur diaphysis. Eighty continuous slices, starting 100 μm from the distal growth plate to the area where both condyles were no longer visible, were selected for analysis. All trabecular bone from each selected slice was segmented for 3D reconstruction (sigma = 1.2, supports = 2 and threshold = 200). Mineral density (bone mineral density, BMD; and bone volume to tissue volume, BV/TV) and trabecular microarchitecture parameters (trabecular number, Tb.N; trabecular thickness, Tb.Th; and trabecular spacing, Tb.Sp) were analyzed using the built-in software.

### Bone mechanical properties

The mechanical properties of the bone and compressive strength of the femur mid-diaphysis and compressive strength were examined by a three-point bending mechanical strength device (Instron 5943, USA), as shown in Figure 5C. The femurs were prepared for biomechanical testing by wrapping in normal saline-soaked gauze and freezing at -20 °C. The fresh-frozen femurs were thawed at room temperature then centred longitudinally, with the anterior surface on the two lower support points spaced 10 mm apart. Materials testing systems (SANS CMT 5304, China) were used to apply a flexion moment in the anteroposterior plane of the surface at a constant displacement rate of 1.5 mm/min until fracture occurred during three-point bending. Mechanical data were acquired at 30 Hz and used to determine the maximum strain, max load and stiffness.

The mechanical properties of the bone and compressive strength of tibia mid-diaphysis and compressive strength were examined by a three-point bending mechanical test system (UniVert, Canada), as shown in Figure 5H and Figure 6E. The tibias were prepared for biomechanical testing by wrapping in normal saline-soaked gauze. The anterior surface on the two lower support points was spaced 8 mm apart. A UniVert mechanical test system was used to apply a flexion moment in the anteroposterior plane of the surface at a constant displacement rate of 0.6 mm/min until 0.5 mm of displacement occurred during three-point bending. The mechanical data were acquired and used to determine the max load, stiffness and elasticity modulus.

### Bone histochemistry

#### Undecalcified bone sections

After euthanasia, femur samples were obtained and fully fixed with 4% PFA. As shown in Figures 1D-E, 4C, 5A-B, and Figures S2B, S2E, S4H-I, the fixed samples were dehydrated in gradient alcohol, cleared with xylene, and embedded in gradient methyl methacrylate (MMA). Sections were acquired using an EXAKT 300CP tissue cutting system and polished to approximately 20-40 μm using an EXAKT 400CS micro grinding system (EXAKT Apparatebau, Germany). As shown in Figures 5F and 6C, the femur samples were fully fixed with 4% PFA, and then embedded directly into optimal cutting temperature (OCT) compound in the cryostat (Leica Microsystems, USA). Then, a series of frozen sections (20 μm) was acquired.

#### Double calcein labeling

To examine the bone formation rate in the mice, calcein (Sigma, USA) was injected (10 mg/kg body weight) twice before euthanasia. After euthanasia, femur samples were isolated and made into undecalcified plastic sections, and these sections were examined with a fluorescence microscope (Nikon, Japan), bone formation rate was analyzed using ImageJ software (NIH, USA).

#### Goldner's trichrome staining

To check new osteoid formation capability of the samples, 20-40 μm of undecalcified sections were prepared with an EXAKT system and stained with Goldner's trichrome stain. All staining procedures were conducted according to standard protocols. In brief, the nuclear counterstain Weigert's haematoxylin was applied, followed by staining with ponceau/acid fuchsin/azophloxine, 0.4% orange G and 0.2% light green in the Goldner's trichrome stain to detect bone and osteoid: black (nuclei blue); green (mineralized bone/muscle); red (osteoid/collagen). The sections were examined under a fluorescence microscope (Osteo-Scan, USA).

#### Immunohistochemical staining

The femur samples were fixed with 4% PFA for 2 days and then embedded in paraffin after two weeks of decalcification with 10% EDTA treatment. Serial 5 μm sections of the femur were made using a microtome, and the femur sections were stained with immunohistochemical staining. Briefly, femur sections were processed for antigen retrieval for 15 min and blocked in 3% BSA for 30 min. The sections were then incubated overnight with primary antibody against osteocalcin (1:2000, Servicebio, China) at 4 °C. After three washes with PBS, the sections were incubated with HRP-conjugated anti-rabbit IgG (1:400, Servicebio, China) for 50 min. A 3N-diaminobenzidine tetrahydrochloride (DAB) kit (Servicebio, China) was used to detect immunoactivity, followed by counterstaining with hematoxylin (Servicebio, China). The sections were examined under a fluorescence microscope (Nikon, Japan).

### Cell culture and transfection

#### Primary osteoblast preparation

Mouse primary osteoblasts were isolated from the femora of 2-month male mice according to a previously published bone research protocol 17. Briefly, femora and tibias were isolated without connective tissue and maintained in α-MEM. The epiphyses at both ends were then cut open, and the bone marrow contents were removed. The cleaned diaphyses were then cut into small pieces (1-2 mm^3^) and washed with PBS. Bone chips were then preincubated in collagenase I solution (α-MEM + 1 mg/mL Type I collagen, 200 rpm) at 37 °C for 2 h (4 times × 30 min each time) for removal of soft tissue and adherent cells. Then, primary osteoblasts were collected from the fifth to seventh incubation for culture.

#### Cell culture

The primary osteoblasts were cultured in α-MEM (Gibco, USA) supplemented with 10% FBS (Corning, USA), 100 µg/mL streptomycin and 100 units/mL penicillin (Amresco, USA). The primary osteoblasts were maintained under standard cell culture conditions with 5% CO_2_ and 95% humidity in a 37 °C incubator, and were not used after passage 6. For the experiments, confluent cells were passaged with 0.25% trypsin containing 10 mM EDTA. For the primary osteoblast differentiation experiment, primary osteoblasts were cultured in a 24-well plate at 1 × 10^5^ cells per well with osteogenic medium containing 10% FBS (Biological Industries, Israel), 100 µg/mL streptomycin, 100 units/mL penicillin, 50 μg/mL ascorbic acid and 10 mM β-glycerophosphate.

The MC3T3-E1 murine osteoblastic cell line was generously provided by Dr. Hong Zhou from the University of Sydney. The MC3T3-E1 cells were cultured in α-MEM supplemented with 10% FBS (Biological Industries, Israel), 100 µg/mL streptomycin, and 100 units/mL penicillin and incubated in a humidified incubator at 37 °C and 5% CO_2_.

#### Cell transfection

Primary osteoblasts were seeded at a cell density of 2 × 10^4^ cells/cm^2^ and transfected with 30 nM antagomir-138-5p (AMO) or 50 nM agomir-138-5p (Ago) by Lipofectamine 2000 (Invitrogen, USA), or 0.5 μg/mL a specially designed MACF1 3'UTR (3'UTR) plasmid by Entranster^TM^ H4000 (Engreen Biosystem, USA). After transfection for 6 h, the serum-free medium was replaced by a growth medium. Cells were harvested for real-time PCR, Western blot analysis and alkaline phosphatase staining after 48 h. The antagomir-138-5p (Gene Pharma, China) sequence was 5'-CGGCCUGAUUCACAACACCAGCU-3'. The agomir-138-5p (Ribobio, China) sequence was 5'- AGCUGGUGUUGUGAAUCAGGCCG-3' (positive-sense strand) and 5'-UCGACCACAACACUUAAUCCGGC-3' (antisense strand). The MACF1 3'UTR plasmid was designed by 6 copies of the *Macf1* 3'UTR sequence which was complementary to miR-138-5p.

### *In vitro* simulation of mechanical stimuli

#### Mechanical unloading (MU)

A desktop random positioning machine (RPM, the Center for Space Science and Applied Research of Chinese Academy of Sciences, China) was used to simulate mechanical unloading conditions for the cell culture, as described previously 14. The cell culture vessel was fixed on the inner frame and the RPM was placed inside a 37 °C incubator. For simulated mechanical unloading studies, primary osteoblasts were seeded at a density of 2 × 10^4^ cells/cm^2^ on glass coverslips in a 90 mm dish and incubated at 37 °C. The cells were cultured or transfected for 12 h. RPM culture flasks filled with growth medium (without air bubbles) were tightly capped and mounted in the inner frame of the RPM. The machine was operated in random mode of speed (0-8 rpm) and direction including both inner and outer frames for 24 or 48 h. The cells in the static control group were cultured in the same 37 °C incubator without rotation.

#### Cyclic stretch (CS)

The Flexcell 4000^TM^ Tension Plus^TM^ unit (Flexcell International Corporation, USA) was used to simulate mechanical cyclic stretch for the cell culture, as described previously 13. Cells were seeded into 6-well flexible silicone rubber BioFlex^TM^ plates coated with collagen type I (Flexcell International Corporation, USA) at a density of 1 × 10^5^ cells/mL in 2 mL medium. The cells were cultured for 12 to 24 h to reach 80% confluency, at which time the growth medium was replaced. A cyclic mechanical tension with a 1.0 Hz sinusoidal curve at 10% deformation was applied for 12 h or 24 h. The cells were incubated in the 37 °C incubator during the stretching. The control group was maintained under identical culture conditions without mechanical cyclic stretch. Cells were collected at the same time point.

### Cell staining

#### Alkaline phosphatase (ALP) staining

Alkaline phosphatase staining was performed with a BCIP/NBT Alkaline Phosphatase Colour Development Kit (Beyotime, China) according to the manufacturer's instructions. Briefly, cells were carefully rinsed with PBS and fixed with neutral buffered formalin (10%) for 15 min. The fixed cells were rinsed with PBS three times again and then BCIP/NBT liquid substrate was added to each well. Finally, the cells were washed with ddH_2_O after the colour turned blue/purple. The stained cell plates or glass coverslips were imaged by a scanner (Canon, Japan).

#### Alizarin red S (ARS) staining

Cells were carefully rinsed with PBS and fixed with neutral buffered formalin (10%) for 15 min. The fixed cells were rinsed with PBS three times and stained with 0.5% alizarin red S (Sigma, USA) solution (pH 4.2) for 15 min at room temperature. After washing with ddH_2_O four times, the cell plates with mineralized nodules were imaged by a scanner (Canon, Japan).

### Western blot analysis

Cells were lysed on ice in Cell Lysis Buffer for Western and IP (Beyotime, China) supplemented with 1% Protease Inhibitor Cocktail Set III (Merck, Germany). Protein fractions were collected by centrifugation at 15,000 × g and 4 °C for 5 min, subjected to 8% SDS-PAGE (MACF1) or 10% SDS-PAGE (GAPDH) and then transferred to PVDF membranes (PALL, USA). The membranes were blocked with 5% skim milk and incubated overnight with specific primary antibodies at 4 °C. HRP-labeled secondary antibody (Beijing CoWin Bioscience, China) was added, and then, the membrane was visualized using a T5200 Multi Chemiluminescence Detection System (Tanon, China) as recommended by the manufacturer. The following primary antibodies were used: anti-MACF1 rabbit polyclonal antibody (1:1000, Abcam, USA) and anti-GAPDH rabbit polyclonal antibody (1:1000, Servicebio, China). Quantification of the protein level was performed using ImageJ software (NIH, USA). The relative MACF1 protein level was normalized to the control or mock group. GAPDH protein level was used as an internal control for MACF1.

### Dual-luciferase reporter assay

The mouse *Macf1* gene (NM_001199136) 3'UTR containing the miR-138-5p-binding sequence was amplified by PCR. The PCR product was subcloned into the XbaI site downstream of the stop codon in a GV306 empty vector (GeneChem, China). Transient transfection of the MC3T3-E1 cells (8 × 10^6^ cells per well) was performed in a 6-well plate with the Neon^®^ Transfection System (Invitrogen, USA) following the manufacturer's instructions. The cells were cotransfected with 100 ng of agomir-138-5p or corresponding negative controls and 8000 ng of luciferase reporter vector. One hundred nanograms of agomir was added to the medium after electroporation. The cells were cultured for 48 h, and luciferase assays were performed with the Dual-Luciferase Reporter Assay System (Promega, USA) according to the manufacturer's instructions. Luminescent signals were quantified by microplate reader (Synergy, USA), and each value for the firefly luciferase constructs was normalized to the values obtained by Renilla luciferase assay.

### miR-138-5p inhibitor (AMO) therapy

#### AMO injection in an unloading-induced osteoporosis mouse model

Three-month-old male BALB/c mice received three consecutive tail vein injections of antagomir-138-5p (AMO, 10 mg/kg body weight) with the osteoblast-targeted delivery system, the negative control antagomir-NC (10 mg/kg body weight) with the osteoblast-targeted delivery system or the osteoblast-targeted delivery system alone every other day before hindlimb unloading. Then, the mice were subjected to hindlimb unloading through tail suspension for 28 days. All mice were injected intraperitoneally with green-fluorescent calcein (10 mg/kg body weight) in a time sequence of 10 days and 2 days before euthanasia.

#### AMO injection and treadmill exercise with osteoblastic miR-138-5p transgenic mice

Two-month-old male TG male mice received three consecutive tail vein injections of antagomir-138-5p (AMO, 10 mg/kg body weight) with the osteoblast-targeted delivery system, antagomir-NC (10 mg/kg body weight) with the osteoblast-targeted delivery system or the osteoblast-targeted delivery system alone for every 10 days. After the first injection, the mice were subjected to the treadmill exercise for 4 weeks as previously described. After the 4-week treadmill exercise period, all the mice were euthanized. All mice were injected intraperitoneally with green-fluorescent calcein (10 mg/kg body weight) in a time sequence of 8 days and 2 days before euthanasia.

#### AMO injection and treadmill exercise in aged mice

Twenty-month-old male C57BL/6 mice received three consecutive tail vein injection of antagomir-138-5p (AMO, 10 mg/kg body weight) with the osteoblast-targeted delivery system, antagomir-NC (10 mg/kg body weight) with the osteoblast-targeted delivery system or the osteoblast-targeted delivery system alone for every 10 days. After first injection, the mice were subjected to the received treadmill exercise for 4 weeks as previously described. After 4 weeks of the treadmill exercise, all the mice were euthanized. All mice were injected intraperitoneally with green-fluorescent calcein (10 mg/kg body weight) in a time sequence of 10 days and 2 days before euthanasia.

#### Statistical analysis

For data consisting of two independent variables (Figure 5, Figure S7), two-way ANOVA was performed to study the interaction between fixed factors, and one-way ANOVA was then used to perform a variance analysis. For data that did not contain two independent variables, each factors were treated as a single variable, and one-way ANOVA was used for a variance analysis. Significance between two groups was determined using Student's *t*-test. For human experiments, all numerical data were expressed as the mean ± sem. For the mouse and cell experiments, all numerical data were expressed as the mean ± sd. GraphPad Prism 6 software and SPSS 25 statistical software were used for the data analysis, and the significance level was set to a 95% confidence interval (*P < 0.05, **P < 0.01).

## Results

### miR-138-5p is a mechanoresponsive miRNA and negatively correlates with bone formation

Reduced mechanical stimuli (unloading or aging) on bones are among the main causes of degenerative osteoporosis. We first detected the expression of the reported mechanosensitive miRNAs in the tibias of hindlimb unloaded mice, and identified that miR-138-5p was the most differentially expressed miRNA (Figure 1A). Moreover, the miR-138-5p level was increased in the bone of the aged mice (Figure S1A). Next, we collected bone specimens from bedridden (age-matched) and elderly (non-bedridden) male osteoporotic patients to confirm that miR-138-5p was a clinical mechanoresponsive miRNA. The real-time PCR data showed that when grouped by the length of the bedridden period, the expression trends of miR-138-5p were positively correlated with the bedridden period (Figure 1B). Osteogenic marker genes (*Alpl* and *Bglap*) expression and BMD (*T*-score) were negatively correlated with the bedridden period. We also found that the linear function of miR-138-5p level and the changes in the osteogenic marker genes and BMD showed a negative correlation (Figure 1C, Figure S1B-C). In addition, in bone specimens from elderly male osteoporotic patients (non-bedridden), the miR-138-5p level was elevated with age, and negatively correlated with expression of the osteogenic marker genes (Figure 1B-C, Figure S1D-E). These data indicate that, reduction of mechanical stimuli may intensify the onset of osteoporosis, and elevate the miR-138-5p level.

To verify the response of miR-138-5p to mechanical stimuli, and to confirm that miR-138-5p is derived from osteoblasts, the hindlimb unloaded mouse model was used. First, primary osteoblasts from hindlimb unloaded mice were isolated, and the miR-138-5p level was detected. The results showed that as unloading time was extended, the miR-138-5p level was significantly increased, accompanied by decreased expression of the osteogenic marker genes (Figure 1D, Figure S2C). In addition, microCT and double calcein labeling showed that after 4 weeks, hindlimb unloading induced osteoporosis in mice (Figure 1D, Figure S2A-B). After hindlimb unloading, the mice were lowered to the ground (reloading period) and we noticed that the expression levels of miR-138-5p and osteogenic marker genes were both partially restored (Figure 1D, Figure S2C). In addition, similar results were obtained with the aged mice, in which bone formation capability decreased with age, and the miR-138-5p level increased in primary aged osteoblasts (Figure 1E, Figure S2D-F). Thus far, we have screened and verified that bone, especially osteoblasts derived miR-138-5p is mechanoresponsive, and the level of miR-138-5p is negatively correlated with bone formation.

### Mechanoresponsive miR-138-5p inhibits osteoblast differentiation

**Figure 2:**
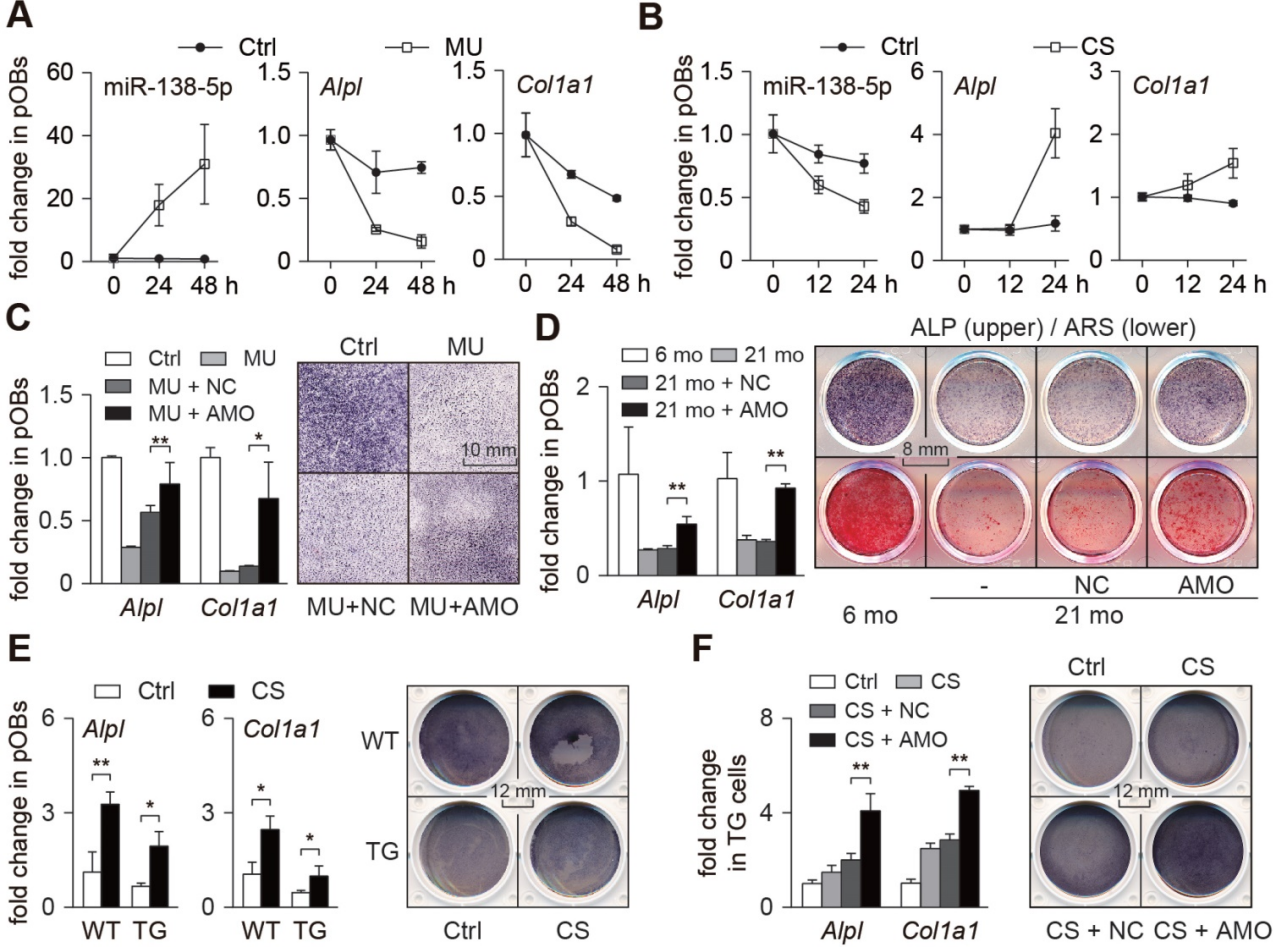
** Mechanoresponsive miR-138-5p inhibits osteoblast differentiation.** (**A**) Real-time PCR analysis of miR-138-5p (left), *Alpl* (middle) and *Col1a1* (right) levels in primary osteoblasts cultured for 0, 24, and 48 h under mechanical unloading (MU) condition, respectively. (**B**) Real-time PCR analysis of miR-138-5p (left), *Alpl* (middle) and *Col1a1* (right) levels in primary osteoblasts cultured for 0, 12, and 24 h under cyclic stretch (CS) condition, respectively. (**C**) Real-time PCR analysis of *Alpl* and *Col1a1* mRNA levels (left) and representative images of ALP staining (right) in primary osteoblasts treated with antagomir-138-5p (AMO) under MU condition. Scale bar, 10 mm. (**D**) Real-time PCR analysis of *Alpl* and *Col1a1* mRNA levels (left), and representative images of ALP staining (right, upper) and Alizarin Red S (ARS) staining (right, lower, 18 d) in primary 21-month aged osteoblasts treated with AMO. Scale bar, 8 mm. (**E**) Real-time PCR analysis of *Alpl* and *Col1a1* mRNA levels (left), and representative images of ALP staining (right) in TG and WT cells cultured under CS condition. Scale bar, 12 mm. TG cells: primary osteoblasts isolated from miR-138-5p transgenic mice. WT cells: primary osteoblasts isolated from WT mice. (**F**) Real-time PCR analysis of *Alpl* and *Col1a1* mRNA levels (left), and representative images of ALP staining (right) in TG cells treated with AMO cultured under CS condition. Scale bar, 12 mm. U6 small nuclear RNA was used as the internal control for miR-138-5p, and *Gapdh* was used as the internal control for mRNAs. n = 3 in each group for Real-time PCR analysis. Data are represented as mean ± s.d. Significances were determined using student's *t*-test between two groups. *P* value less than 0.05 was considered significant in all cases (*P < 0.05, **P < 0.01).

To verify whether miR-138-5p is mechanoresponsive in osteoblasts* in vitro*, a Random Positioning Machine (RPM) and Flexcell 4000 Stretch System were adopted to simulate mechanical unloading (MU) conditions and cyclic stretch (CS) mechanical loading conditions *in vitro*, respectively. We isolated primary osteoblasts from 2-month-old wild-type male mice to estimate their differentiation ability under different degrees of mechanical unloading or loading conditions. We found that the miR-138-5p level was continually increased in these primary osteoblasts under mechanical unloading conditions, and the osteogenic differentiation capability of osteoblasts was increased (Figure 2A and Figure S3A). Inversely, the miR-138-5p level was continually decreased in primary osteoblasts under mechanical loading condition, accompanied by increased osteoblast differentiation capability (Figure 2B and Figure S3B). Taken together, these results confirm miR-138-5p as a mechanoresponsive miRNA with expression that is negatively correlated with osteoblast differentiation* in vitro*.

We then studied the role of miR-138-5p in osteoblast differentiation* in vitro*. Primary osteoblasts transfected with miR-138-5p antagonist (AMO) were found to have elevated expression of osteogenic marker genes and an enhanced capability to form mineralized nodules (Figure S4A), while primary osteoblasts transfected with miR-138-5p agonist (Ago) showed decreased expression of osteogenic marker genes and reduced formation of mineralized nodules (Figure S4B). Further, we constructed an osteoblast-specific miR-138-5p transgenic (TG) mouse model using the runt-related transcription factor 2 (Runx2) promoter (Figure S4C-D), and found lower expression of osteogenic marker genes, lower ALP activity and decreased capability to form mineralized nodules in the primary osteoblasts of the TG mice compared to those of the WT mice (Figure S4E-F). The TG mice also had lower bone mass and bone mineral density, deteriorated trabecular microarchitecture (Figure S4G), and decreased bone formation ability (Figure S4H-J). These data indicate that miR-138-5p inhibits osteoblast differentiation and bone formation.

To further verify whether mechanical stimuli regulate osteoblast differentiation through miR-138-5p, both mechanical unloading and mechanical loading experiments were conducted. For the mechanical unloading experiment, primary osteoblasts were treated with miR-138-5p antagonist (AMO) and then cultured under mechanical unloading conditions. Compared with the control group, osteogenic marker genes (*Alpl* and *Col1a1*) expression and ALP activity were decreased in the mechanical unloading group, while the decrease in osteogenic marker genes induced by mechanical unloading was restored by treatment with the miR-138-5p antagonist (Figure 2C). Similarly, the decrease of osteoblast differentiation in the primary osteoblasts isolated from the aged mice was also counteracted by treatment with the miR-138-5p antagonist (Figure 2D). In the mechanical loading experiment, we found that mechanical loading increased osteoblast differentiation capability. However, compared with the WT osteoblasts, the increase in the TG cells was limited (Figure 2E). Because of the partial deficiency of the TG cells to respond to mechanical loading due to the high level of miR-138-5p, the miR-138-5p antagonist was used to silence miR-138-5p expression in TG cells, and the cells were then cultured under mechanical loading conditions. The osteoblast differentiation capability was significantly upregulated after treatment with the miR-138-5p antagonist in TG cells under CS mechanical loading conditions (Figure 2F). These data suggest that the regulation of osteoblast differentiation by mechanical stimuli partly depends on mechanoresponsive miR-138-5p.

### Mechanoresponsive miR-138-5p directly targets MACF1 to inhibit osteoblast differentiation

**Figure 3:**
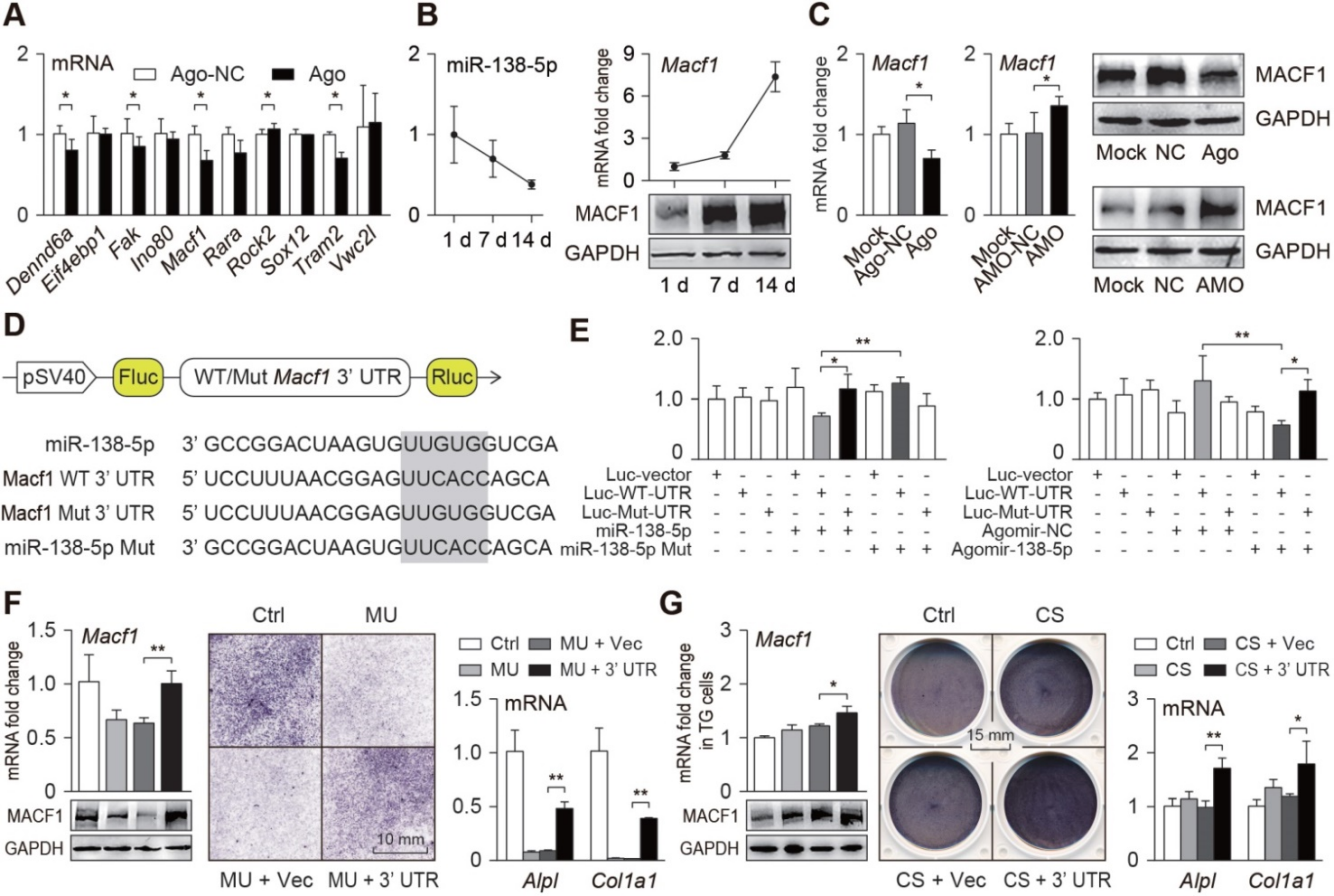
** miR-138-5p directly targets MACF1 to regulate osteoblast differentiation.** (**A**) Real-time PCR analysis of candidate target genes expressions after primary osteoblasts treated with miR-138-5p agonist (Ago). (**B**) Relative expression pattern of miR-138-5p and MACF1 in primary osteoblasts during osteo-induction. (**C**) Real-time PCR and Western Blot analysis of MACF1 expression in primary osteoblasts treated with either miR-138-5p agonist (Ago) or antagonist (AMO), respectively. (**D**) Schematic graph showing the design of dual-luciferase reporters with either *Macf1* WT 3'UTR or *Macf1* mutant 3'UTR. The sequences of miR-138-5p and the mutant miR-138-5p (miR-138-5p Mut) are shown. Fluc, firefly luciferase; Rluc, Renilla luciferase. (**E**) The effects of miR-138-5p recombinant plasmid (miR-138-5p) or mutated control (miR-138-5p Mut) on luciferase activity of *Macf1* WT 3'UTR reporter (Luc-WT-UTR), *Macf1* mutant 3'UTR reporter (Luc-Mut-UTR), or luciferase vector control (Luc-vector) in MC3T3-E1 cells, respectively (left).The effects of agomir-138-5p on luciferase activity of Luc-WT-UTR, Luc-Mut-UTR, or Luc-vector in MC3T3-E1 cells, respectively (right). (**F**) Real-time PCR (upper, left) and Western Blot (lower, left) analysis of MACF1 expressions, and representative images of ALP staining (middle) and Real-time PCR analysis of *Alpl* and *Col1a1* (right) levels in primary osteoblasts treated with *Macf1* 3'UTR plasmid or blank vector under MU condition, respectively. Scale bar, 10 mm. (**G**) Real-time PCR (upper, left) and Western Blot (lower, left) analysis of MACF1 expressions, and representative images of ALP staining (middle) and Real-time PCR analysis of *Alpl* and *Col1a1* (right) levels in TG cells treated with *Macf1* 3'UTR plasmid or blank vector under CS condition. Scale bar, 15 mm. *Gapdh* was used as the internal control for mRNAs. n = 3 in each group for Real-time PCR analysis. Data are represented as mean ± s.d. Significances were determined using student's *t*-test between two groups. *P* value less than 0.05 was considered significant in all cases (*P < 0.05, **P < 0.01).

To explore the mechanism by which miR-138-5p regulates osteoblast differentiation, we screened and verified potential targets of miR-138-5p. First, potential targets of miR-138-5p were predicted using the TargetScan database, and a dozen potential targets related to osteogenic differentiation were obtained. To verify this hypothesis, we treated primary osteoblasts with the miR-138-5p agonist and found multiple differentially expressed genes, such as *Macf1* (Figure 3A). During osteoblast differentiation of the primary osteoblasts, the miR-138-5p level was continuously decreased, while the expression of MACF1 continuously increased (Figure 3B, Figure S5B). When osteoblasts were transfected with miR-138-5p agonist or antagonist, MACF1 expression was inhibited or promoted, respectively (Figure 3C, Figure S5C). Therefore, we propose that MACF1 may be a potential downstream target of miR-138-5p. To verify this interaction, the targeted binding regions of miR-138-5p and the *Macf1* 3'UTR, which is highly conserved among mammalian species, were obtained from the TargetScan database (Figure S5A). After cloning the binding region or its mutant sequence into a dual luciferase reporter vector (Figure 3D), we found that the luciferase activity was significantly decreased in the osteoblasts simultaneously transfected with the miR-138-5p overexpression plasmid and the WT *Macf1* 3' UTR plasmid, while mutation of neither miR-138-5p nor the *Macf1* 3'UTR changed the luciferase activity significantly. In addition, similar results were obtained after osteoblasts were transfected with miR-138-5p agonist (Figure 3E). These data suggest that miR-138-5p directly targets MACF1.

To verify this interaction under different mechanical conditions, we constructed an overexpression plasmid containing repetitive sequences of the *Macf1* 3'UTR targeted region (3'UTR), and transfected it into primary osteoblasts, which were then subjected to mechanical unloading. We found that the repetitive sequences of the 3'UTR could counteracted the inhibitory effect of mechanical unloading or aging on the MACF1 expression and osteoblast differentiation (Figure 3F, Figure S5D-E). In addition, transfection of TG osteoblasts carrying the 3'UTR greatly accelerated the effect of the mechanical loading on MACF1 expression and osteoblast differentiation (Figure 3G, Figure S5F). These data verify that MACF1 is a key target of miR-138-5p for sensing different mechanical stimuli and regulating osteoblast differentiation.

### Osteoblast-targeted delivery of miR-138-5p antagonist restores mechanical unloading-induced osteoporosis

**Figure 4:**
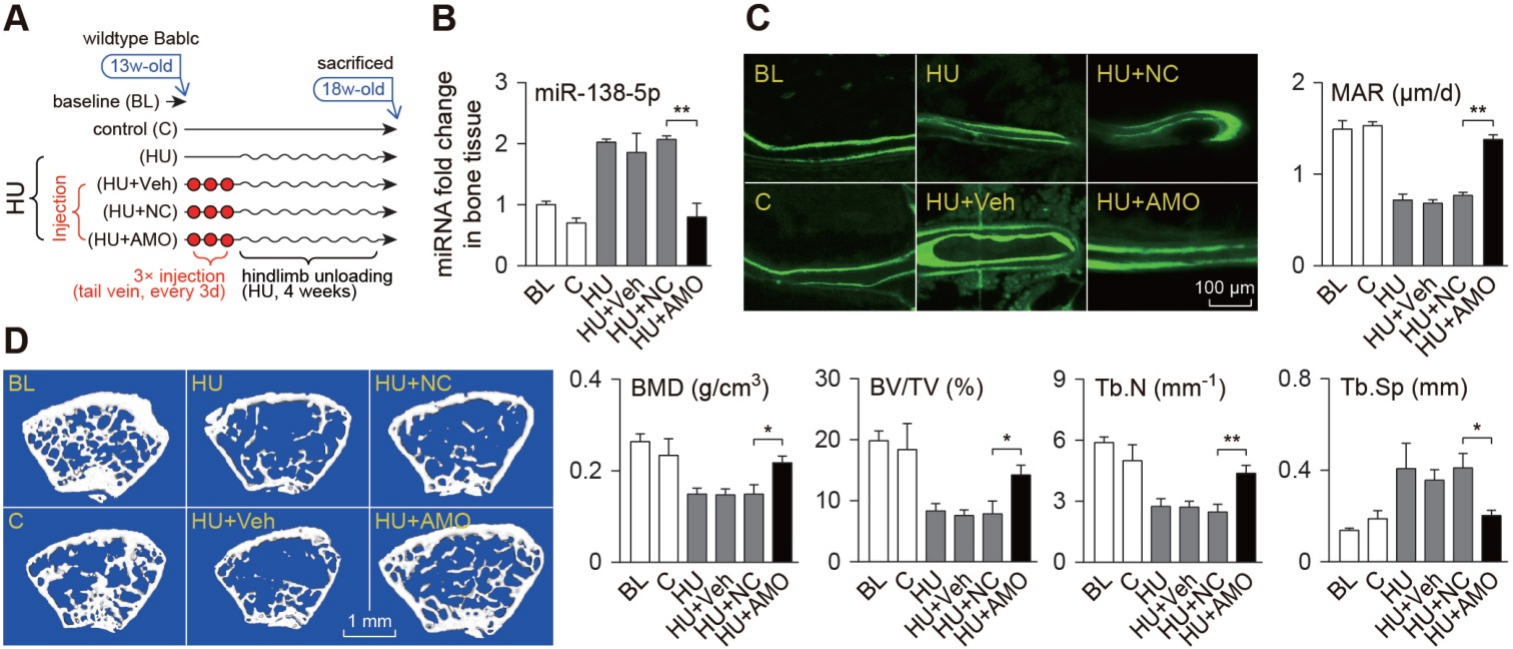
** Bone-targeted delivery of miR-138-5p antagonist restores unloading-induced osteoporosis.** (**A**) Schematic graph showing experiment grouping and miR-138-5p antagonist (AMO) treatment of hindlimb unloaded mice. Mice were injected with AMO three times before 4-week hindlimb unloading. BL (baseline, 3-month-old mice), C (4-month-old mice, without any injection), HU (4-month-old mice, hindlimb unloading for 28 d without any injection), HU + Veh (4-month-old mice, hindlimb unloading for 28 d and injected with osteoblast-targeted delivery system), HU + NC (4-month-old mice, hindlimb unloading for 28 d and injected with both osteoblast-targeted delivery system and antagomir-NC), HU + AMO (4-month-old mice, hindlimb unloading for 28 d and injected with both osteoblast-targeted delivery system and antagomir-138-5p). (**B**) Real-time PCR analysis of miR-138-5p relative level in bone tissue of hindlimb unloaded mice treated with AMO. n = 4 for each group. (**C**) Representative calcein double labeling images (left) and dynamic histomorphometric analysis of MAR (right) showing bone formation capacity in hindlimb unloaded mice pretreated with AMO. MAR, mineral apposition rate. Scale bar, 100 µm. n ≥ 5 mice for each group. (**D**) Representative 3D reconstruction images showing microarchitecture(left), and microCT statistical analysis of BMD, BV/TV, Tb.N, Tb.Sp (right) in distal femur of hindlimb unloaded mice pretreated with AMO. BMD, bone mineral density; BV/TV, bone volume fraction; Tb.N, trabecular number; Tb.Sp, trabecular spacing. Scale bar, 1 mm. n ≥ 5 mice for each group. U6 small nuclear RNA was used as the internal control for miR-138-5p. Data are represented as mean ± s.d. Significances were determined using student's *t*-test between two groups. *P* value less than 0.05 was considered significant in all cases (*P < 0.05, **P < 0.01).

To verify the therapeutic effect of bone-targeted inhibition of miR-138-5p on osteoporosis induced by mechanical unloading, mice preinjected with the bone-targeted miR-138-5p antagonist (AMO) were subjected to hindlimb suspension for 4 weeks (Figure 4A). We found that the miR-138-5p level in the mouse bones was increased significantly after unloading, but it was decreased to a normal level after AMO treatment (Figure 4B). The calcein-labeled bone formation experiment showed that the bone formation rate decreased after hindlimb unloading, but it was restored to nearly the normal level by AMO treatment (Figure 4C). Further, we scrutinized bone mass and trabecular microarchitecture and found that after AMO treatment, the bone mineral density and bone mass in the unloaded mice increased significantly compared to the density and mass in the controls, and trabecular microarchitecture deterioration was alleviated (Figure 4D, Figure S6C). In addition, the decrease in *Macf1* and osteogenic marker gene levels in bone tissue of the hindlimb unloaded mice was also restored by AMO treatment (Figure S6A-B). These data suggest that bone-targeted inhibition of miR-138-5p prevented osteoporosis induced by mechanical unloading.

### Osteoblast-targeted delivery of miR-138-5p antagonist sensitizes bone anabolic action to mechanical stimuli in miR-138-5p transgenic mice

**Figure 5:**
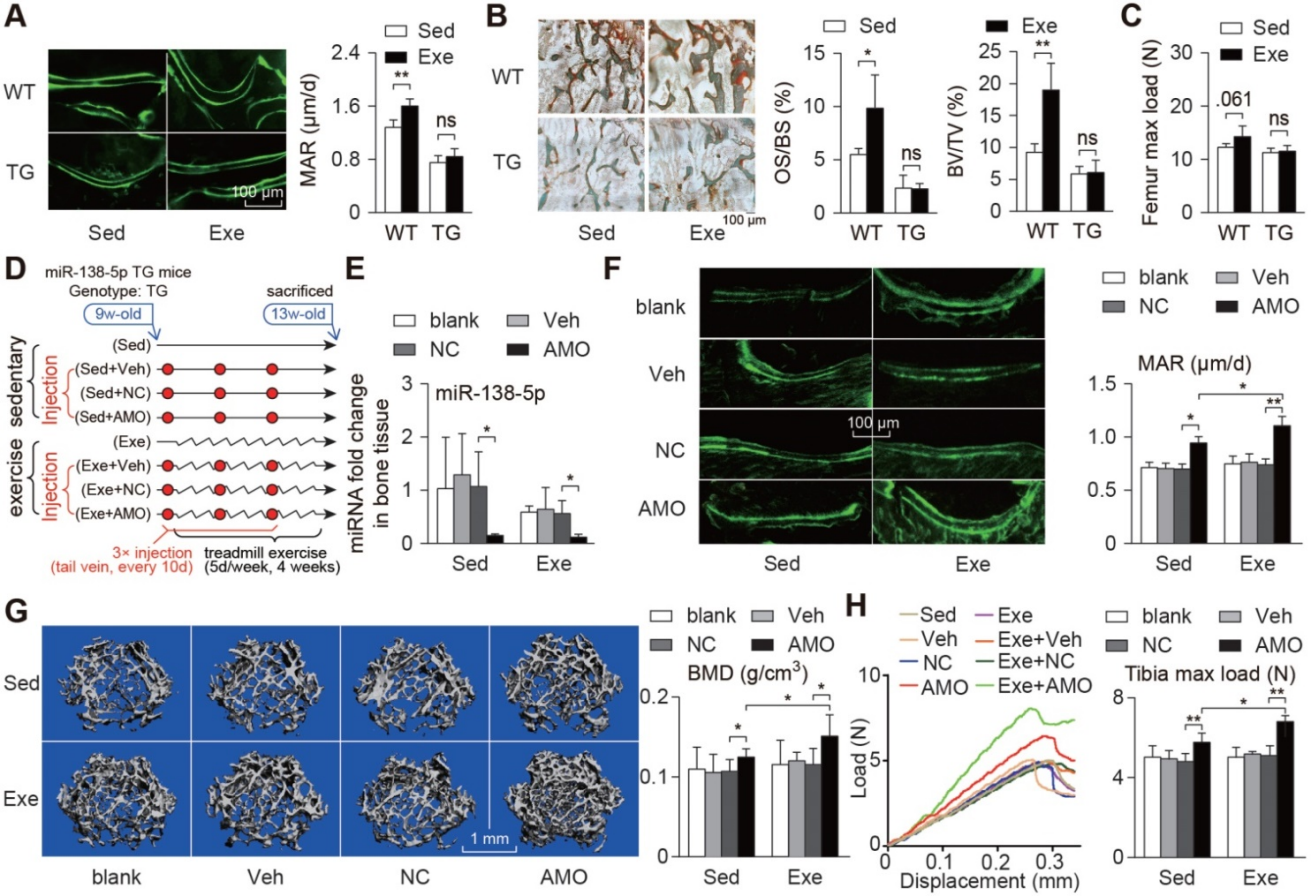
** Bone-targeted delivery of miR-138-5p antagonist sensitizes anabolic bone action to mechanical stimuli in miR-138-5p transgenic mice.** (**A**) Representative calcein double labeling images and dynamic histomorphometric analysis of MAR showing bone formation capacity in distal femur of miR-138-5p transgenic (TG) mice and wild type (WT) mice after 4-week treadmill exercise. Scale bar, 100 µm. MAR, mineral apposition rate. Sed, sedentary, Exe, exercise. n ≥ 5 mice for each group. (**B**) Representative images of osteoid formation indicated by Goldner's trichrome staining in distal femur of TG and WT mice after 4-week treadmill exercise. Scale bar, 100 µm. Statistical analysis of osteoid parameters (OS/BS) and trabecular parameters (BV/TV) in distal femur of all groups. OS/BS: osteoid surface per bone surface; BV/TV: Bone volume per tissue volume; n = 4 for each group. (**C**) Three-point bending measurement of femur max load in WT and TG mice after treadmill exercise. n ≥ 5 mice for each group. (**D**) Schematic graph showing experiment grouping, treadmill exercise and miR-138-5p antagonist (AMO) treatment in 2-month male TG mice. Mice were injected with AMO three times during 4-week treadmill exercise. blank (without any injection), Veh (injected with osteoblast-targeted delivery system), NC (injected with both osteoblast-targeted delivery system and antagomir-NC), AMO (injected with both osteoblast-targeted delivery system and antagomir-138-5p). (**E**) Real-time PCR analysis of miR-138-5p relative level in bone tissue of TG mice after treadmill exercise and AMO treatment. n = 4 for each group. (**F**) Representative calcein double labeling images and dynamic histomorphometric analysis of MAR showing bone formation capacity in TG mice after treadmill exercise and AMO treatment. Scale bar, 100 µm. n = 6 for each group. (**G**) Representative 3D reconstruction images showing microarchitecture, and microCT analysis of BMD in distal femur after treadmill exercise and AMO treatment. BMD, bone mineral density. Scale bar, 1 mm. n = 6 for each group. (**H**) Representative load-deflection curves for the respective groups, and three-point bending measurement of tibia max load in TG mice after treadmill exercise and AMO treatment. n = 6 for each group. U6 small nuclear RNA was used as the internal control for miR-138-5p. Data are represented as mean ± s.d. Significances were determined using student's *t*-test between two groups. *P* value less than 0.05 was considered significant in all cases (*P < 0.05, **P < 0.01).

To assess the mechanical response of bone tissue in the WT and TG mice, a treadmill exercise was used to simulate the effects of mechanical loading on the mice *in vivo*. After the 4-week treadmill exercise period, the mineral apposition rate (MAR) and osteoid formation of the WT mice were clearly increased compared to those of the control group mice. Nevertheless, the MAR and osteoid formation in the TG mice were only slightly increased without significant differences after the treadmill exercise (Figure 5A-B, Figure S7A). In addition, the increase in bone mechanical strength (maximum strain, stiffness and Max load) was also lower in the TG mice than in the WT mice (Figure 5C, Figure S7B), which revealed that high miR-138-5p levels in bone led to the loss of mechanical response to mechanical loading in the TG mice.

To study the therapeutic effect of bone-targeted inhibition of miR-138-5p or mechanical stimuli on osteoporosis induced by high miR-138-5p levels, TG mice were injected with AMO during the 4-week treadmill exercise period (Figure 5D). We found that the AMO injection significantly suppressed the miR-138-5p levels and increased the bone formation rate and bone mass and improved the trabecular microarchitecture and tibia mechanical strength. In addition, treadmill exercise slightly suppressed miR-138-5p levels and increased the bone formation rate compared with the levels and rate of the sedentary group (Figure 5E-H, Figure S7C-D). Interestingly, in the exercise group injected with AMO, the increases in bone formation rate, bone mass and mechanical strength and the improvement in trabecular microarchitecture were even greater than those of the group treated with either AMO injection or subjected to treadmill exercise (Figure 5E-H, Figure S7C-F). These results suggest that miR-138-5p AMO and running exercise have synergistic effects in antagonizing osteoporosis, and inhibiting miR-138-5p during exercise would greatly benefit osteoporosis treatment.

### Osteoblast-targeted delivery of miR-138-5p antagonist sensitizes bone anabolic action to mechanical stimuli in aged mice

**Figure 6:**
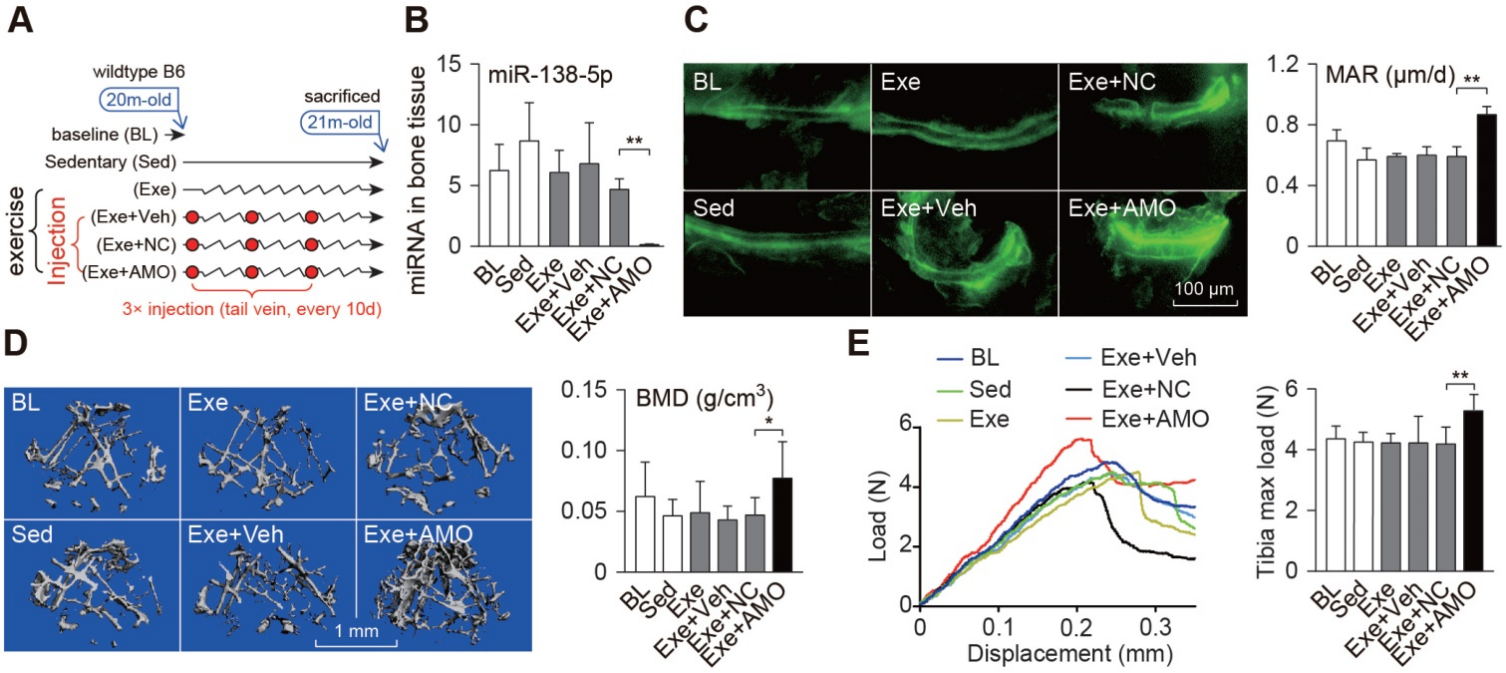
** Bone-targeted delivery of miR-138-5p antagonist sensitizes anabolic bone action to mechanical stimuli in aged mice**. (**A**) Schematic graph showing experiment grouping, treadmill exercise and miR-138-5p antagonist (AMO) treatment in aged mice. Mice were injected with AMO three times during 4-week treadmill exercise. BL (baseline, 20-month-old mice), Sed (21-month-old mice, without any injection and without treadmill), Exe (21-month-old mice, treatment with treadmill exercise and without any injection ), Exe + Veh (21-month-old mice, treatment with treadmill exercise and injected with osteoblast-targeted delivery system), Exe + NC (21-month-old mice, treatment with treadmill exercise and injected with osteoblast-targeted delivery system and antagomir-NC), Exe + AMO (21-month-old mice, treatment with treadmill exercise and injected with osteoblast-targeted delivery system and antagomir-138-5p). (**B**) Real-time PCR analysis of miR-138-5p relative level in bone tissue of aged mice after 4-week treadmill exercise and AMO treatment. n = 4 for each group. (**C**) Representative calcein double labeling images (left) and dynamic histomorphometric analysis of MAR (right) showing bone formation capacity in aged mice after 4-week treadmill exercise and AMO treatment. MAR, mineral apposition rate. Scale bar, 100 µm. n ≥ 5 mice for each group. (**D**) Representative 3D reconstruction images showing microarchitecture (left), and microCT statistical analysis of BMD in distal femur after 4-week treadmill exercise and AMO treatment. BMD, bone mineral density. Scale bar, 1 mm. n ≥ 5 mice for each group. (**E**) Representative load-deflection curves for the respective groups, and three-point bending measurement of tibia max load in aged mice after 4-week treadmill exercise and AMO treatment. n ≥ 5 mice for each group. U6 small nuclear RNA was used as the internal control for miR-138-5p. Data are represented as mean ± s.d. Significances were determined using student's *t*-test between two groups. *P* value less than 0.05 was considered significant in all cases (*P < 0.05, **P < 0.01).

Senile osteoporosis is a common disease with clinical features of degenerative diseases, and is caused by many factors including reduced mechanical stimuli. We verified the therapeutic effects of bone-targeted inhibition of miR-138-5p and mechanical stimuli in senile osteoporosis (Figure 6A). We found that the miR-138-5p levels in the bone tissue of aged mice was not significantly decreased (Figure 6B), and the bone formation rate, bone mass and tibia mechanical strength were also not significantly restored after treadmill exercise (Figure 6C-E, Figure S8A-D), suggesting that simply increasing mechanical stimuli is not effective for the treatment of senile osteoporosis. However, in the treadmill exercise group that received the AMO injection, the miR-138-5p levels decreased significantly, and the bone formation rate increased greatly compared with either exercise group or AMO injection group; additionally, the bone mass, mineral density, trabecular microarchitecture, tibia mechanical strength and expression of *Macf1* and osteogenic marker genes also increased in the combined treadmill exercise and AMO injection group (Figure 6C-E, Figure S8A-D). These results suggest that the mechanosensitivity of bone is decreased in aged mice, but inhibition of miR-138-5p can increase mechanosensitivity to promote bone formation and counteract senile osteoporosis.

In conclusion, these three treatment experiments showed that mechanical stimuli regulate bone formation through miR-138-5p, and can be used to counteract osteoporosis to some extent, but combining mechanical stimuli with bone-targeted inhibition of miR-138-5p greatly increases the effectiveness of this strategy in treating disuse and senile osteoporosis.

## Discussion

In this study, we first identified a clinical mechanoresponsive miRNA, miR-138-5p, that directly targeted MACF1 to inhibit osteoblast differentiation and bone formation. More importantly, we found that miR-138-5p inhibitor can act as a mechanical sensitizer to increase bone anabolic actions in response to mechanical stimuli in osteoporotic mice, which provides a new mechanism and a potential therapeutic strategy for disuse and senile skeletal disorders.

Several studies have shown that changes in mechanical stimuli are significantly correlated with miRNA levels, and many miRNAs are involved in regulating osteogenic differentiation or bone formation 9-13, 15, 16, 18-35. We first screened previously reported mechanosensitive miRNAs in hindlimb unloaded mice and preliminarily identified that miR-138-5p was the most differentially expressed miRNA under mechanical unloading conditions (Figure 1A). Therefore, we chose miR-138-5p as the subject of our subsequent experiments. However, mechanoresponsive miRNAs that contribute to human skeletal disused diseases, such as bedridden or aging osteoporosis, have yet to be identified. In this study, in bone specimens from bedridden and aged male patients, we found that the miR-138-5p level was increased with bedridden time or age and negatively correlated with osteogenic marker gene levels. These observations provide the first clinical insight into the contribution of a mechanoresponsive miRNA to the pathophysiological regulation of bone formation.

Previous studies also mentioned the relationship between miR-138-5p and mechanical stimuli. In osteo-induced bone marrow stromal cells (BMSCs) subjected to extracorporeal shock wave (ESW) 36 and cyclic stretch conditions 37, the expression of miR-138-5p was downregulated significantly while the capability of osteogenic differentiation was increased 36-38. However, most studies of mechanosensitive miRNAs involved in mechanical stimuli regulating bone anabolic activity have not been systematically depicted and illustrate only the roles of mechanosensitive miRNAs under specific conditions, such as *in vitro* or *in vivo* experiments 9, 10. In the present study, we proposed that miR-138-5p was significantly correlated with the level of different mechanical stimuli, and gain-of-function and loss-of-function studies systematically demonstrated the negative roles of mechanoresponsive miR-138-5p in osteogenesis. In addition, the results of this study showed that ALP expression and activity were enhanced by antagomir-NC compared with the mock treatment. The sequence of antagomir-NC or small nucleic acids may be beneficial to osteoblast differentiation, which is a topic that needs to be investigated in the future.

Recently, miR-138 has been reported to play important roles in physiological biological processes in different bone cells (e.g., proliferation 39, apoptosis 40, differentiation 37 and invasion 41), and to serve as a crucial regulator in pathological bone diseases, such as osteoporosis 38, osteosarcoma 42, multiple myeloma 43, osteoarthritis 44, and chondrosarcoma 45. Previously, miR-138 was reported to negatively regulate osteogenic differentiation and ectopic bone formation 23, 36-38, 43, 46-48. For therapeutic consideration, these studies engrafted MSCs pretreated with miR-138-5p inhibitor into immunocompromised mice 23, 38, 43. Here, we found that miR-138-5p was negatively correlated with bone formation in both humans and mice, and the miR-138-5p inhibitor delivered by an osteoblast-targeted delivery system rescued the decrease of bone formation in the hindlimb unloaded and aged mice, which are common osteoporotic mouse models. These findings suggest the use of miR-138-5p as a therapeutic target and the inhibition of miR-138-5p as a pharmacological therapeutic approach for osteoporosis.

Exploration of the downstream targets of miR-138-5p led to the discovery of microtubule actin crosslinking factor 1 (MACF1). Our previous data show that MACF1 positively regulates osteoblast differentiation and bone formation by activating the Wnt/β-catenin or BMP signaling pathways 49-52, which are also likely involved in miR-138 regulation of osteogenic differentiation 47, 48. In addition, MACF1, as a cytoskeleton crosslinker, is negatively correlated with age and also mechanoresponsive under different mechanical conditions 49, 52-55. We also found that the transfection of the *Macf1* 3'UTR repeated sequence showed effects similar to those of the miR-138-5p inhibitor. These results provide a potential mechanism for the involvement of miR-138-5p in mechanosensibility.

Mechanical stimuli promote bone anabolic actions, which is one of the most important countermeasures for osteoporosis 56. Subburaman *et.al* discovered that the conditional knockout of the miR 17-92 cluster in osteoblasts reduced periosteal bone formation and bone anabolic response to exercise 57. Upon deletion of miR-21, the alveolar bone anabolic response to mechanical force in teeth was not affected under normal conditions, but was reduced under orthodontic force 58. In our study, we verified that the bone anabolic activity became insensitive to mechanical stimuli when miR-138-5p levels were elevated in miR-138-5p transgenic mice and aged mice. However, bone-targeted inhibition of miR-138-5p restored the response of bone to mechanical stimuli, and thus led to recovered bone formation capability and bone mass. This outcome may explain why mechanical stimuli are ineffective for bone formation when the miR-138-5p level is elevated and suggests that miR-138-5p inhibitor is a potential mechanical sensitizer for the countermeasure of osteoporosis.

In addition, we discovered that the therapeutic effects of miR-138-5p inhibitor were better than mechanical loading (such as treadmill exercise), but weaker than the combination of miR-138-5p inhibitor and mechanical loading. Long-term bedridden and aged patients lack exercise and receive less mechanical stimuli to the bone 4, 5, 59, 60. Our data suggest that, when additional mechanical stimuli are infeasible (for example, in handicapped and paralyzed patients), miR-138-5p inhibitor treatment alone could effectively restore bone mass. When additional mechanical stimuli are feasible (for example, in a space crew), bone-targeted inhibition of miR-138-5p could greatly improve the promotion of bone formation by mechanical stimuli. More importantly, for those who do not exercise often (for example, in elderly individuals), miR-138-5p inhibitor treatment combined with treadmill exercise shows great therapeutic effects. For the growing elderly population, this combination effectively protects against osteoporosis and alleviates joint and muscle damage caused by exercise. Admittedly, this study focused only on the treadmill exercise model, and the effectiveness of other types of mechanical stimuli still needs further investigation. Moreover, our results also suggest that the background level of miR-138-5p could be used to categorize osteoporotic patients for personalized countermeasures, but this possibility needs further study.

In summary, this study identified a mechanoresponsive miRNA, miR-138-5p, from clinical bone specimens, and *in vitro* and *in vivo* experiments demonstrated that bone-targeted inhibition of miR-138-5p could sensitize the bone anabolic actions to mechanical stimuli, which may provide a potential therapeutic strategy for disuse or senile osteoporosis.

## Abbreviations

Ago: miR-138-5p agonist
ALP/Alpl: alkaline phosphatase
AMO: miR-138-5p antagonist
ARS: alizarin red staining
Bglap: osteocalcin
BMD: bone mineral density
BMP: bone morphogenetic protein
BMSCs: bone marrow stromal cells
BS/BV: bone surface to bone volume
BV/TV: bone volume to tissue volume (bone volume fraction)
Col1a1: collagen type 1 alpha-1
CS: cyclic stretch
Ctrl: control
EDTA: ethylene diamine tetraacetic acid
ESW: extracorporeal shock wave
Exe: exercise
FBS: foetal bovine serum
Gapdh: glyceraldehyde3-phosphatedehydrogenase
h: hour
HU: hindlimb unloading
Luc: luciferase
MACF1: microtubule actin crosslinking factor 1
MAR: mineral apposition rate
microCT: micro-computed tomography
min: minute
miRNA: microRNA
MMA: MethylMethacrylate
MSCs: Mesenchymal stem cells
MU: mechanical unloading
Mut: mutation
NC: negative control
O.Th: osteoid thickness
OCN: osteocalcin
OS/BS: osteoid surface to bone surface
OV/BV: osteoid volume to bone volume
PCR: polymerase chain reaction
PFA: paraformaldehyde
RPM: Random Positioning Machine
Runx2: runt-related transcription factor 2
Sed: sedentary
Tb.N: trabecular number
Tb.Sp: trabecular spacing
Tb.Th: trabecular thickness
TG: transgenic
UTR: untranslated region
Vec: vector
Veh: vehicle (osteoblast-targeted delivery system)
WT: wild type

## References

[1] Huiskes R, Ruimerman R, van Lenthe GH, Janssen JD (2000). Effects of mechanical forces on maintenance and adaptation of form in trabecular bone. Nature.

[2] Zhou T, Gao B, Fan Y, Liu Y, Feng S, Cong Q (2020). Piezo1/2 mediate mechanotransduction essential for bone formation through concerted activation of NFAT-YAP1-ss-catenin. Elife.

[3] Carina V, Della Bella E, Costa V, Bellavia D, Veronesi F, Cepollaro S (2020). Bone's Response to Mechanical Loading in Aging and Osteoporosis: Molecular Mechanisms. Calcif Tissue Int.

[4] Rittweger J, Beller G, Armbrecht G, Mulder E, Buehring B, Gast U (2010). Prevention of bone loss during 56 days of strict bed rest by side-alternating resistive vibration exercise. Bone.

[5] Javaheri B, Pitsillides AA (2019). Aging and Mechanoadaptive Responsiveness of Bone. Curr Osteoporos Rep.

[6] Bartel DP (2018). Metazoan MicroRNAs. Cell.

[7] Gebert LFR, MacRae IJ (2019). Regulation of microRNA function in animals. Nat Rev Mol Cell Biol.

[8] Ha M, Kim VN (2014). Regulation of microRNA biogenesis. Nat Rev Mol Cell Biol.

[9] Yuan Y, Zhang L, Tong X, Zhang M, Zhao Y, Guo J (2017). Mechanical Stress Regulates Bone Metabolism Through MicroRNAs. J Cell Physiol.

[10] Chen Z, Zhang Y, Liang C, Chen L, Zhang G, Qian A (2017). Mechanosensitive miRNAs and Bone Formation. Int J Mol Sci.

[11] Hu Z, Zhang L, Wang H, Wang Y, Tan Y, Dang L (2020). Targeted silencing of miRNA-132-3p expression rescues disuse osteopenia by promoting mesenchymal stem cell osteogenic differentiation and osteogenesis in mice. Stem Cell Res Ther.

[12] Li J, Hu C, Han L, Liu L, Jing W, Tang W (2015). MiR-154-5p regulates osteogenic differentiation of adipose-derived mesenchymal stem cells under tensile stress through the Wnt/PCP pathway by targeting Wnt11. Bone.

[13] Zuo B, Zhu J, Li J, Wang C, Zhao X, Cai G (2015). microRNA-103a functions as a mechanosensitive microRNA to inhibit bone formation through targeting Runx2. J Bone Miner Res.

[14] Chen Z, Zhang Y, Zhao F, Yin C, Yang C, Wang X (2020). Recombinant Irisin Prevents the Reduction of Osteoblast Differentiation Induced by Stimulated Microgravity through Increasing beta-Catenin Expression. Int J Mol Sci.

[15] Li D, Liu J, Guo B, Liang C, Dang L, Lu C (2016). Osteoclast-derived exosomal miR-214-3p inhibits osteoblastic bone formation. Nat Commun.

[16] Wang X, Guo B, Li Q, Peng J, Yang Z, Wang A (2013). miR-214 targets ATF4 to inhibit bone formation. Nat Med.

[17] Bakker AD, Klein-Nulend J (2012). Osteoblast isolation from murine calvaria and long bones. Methods Mol Biol.

[18] Hu Z, Wang Y, Sun Z, Wang H, Zhou H, Zhang L (2015). miRNA-132-3p inhibits osteoblast differentiation by targeting Ep300 in simulated microgravity. Sci Rep.

[19] Ibrahim M, Mohan S, Xing MJ, Kesavan C (2016). Conditional Knockout of the MicroRNA 17-92 Cluster in Type-I Collagen-Expressing Cells Decreases Alveolar Bone Size and Incisor Tooth Mechanical Properties. Folia Biol (Praha).

[20] Li CJ, Cheng P, Liang MK, Chen YS, Lu Q, Wang JY (2015). MicroRNA-188 regulates age-related switch between osteoblast and adipocyte differentiation. J Clin Invest.

[21] Ling S, Zhong G, Sun W, Liang F, Wu F, Li H (2017). Circulating microRNAs Correlated with Bone Loss Induced by 45 Days of Bed Rest. Front Physiol.

[22] Wang H, Sun Z, Wang Y, Hu Z, Zhou H, Zhang L (2016). miR-33-5p, a novel mechano-sensitive microRNA promotes osteoblast differentiation by targeting Hmga2. Sci Rep.

[23] Yan J, Zhang C, Zhao Y, Cao C, Wu K, Zhao L (2014). Non-viral oligonucleotide antimiR-138 delivery to mesenchymal stem cell sheets and the effect on osteogenesis. Biomaterials.

[24] Feng L, Zhang JF, Shi L, Yang ZM, Wu TY, Wang HX (2020). MicroRNA-378 Suppressed Osteogenesis of MSCs and Impaired Bone Formation via Inactivating Wnt/beta-Catenin Signaling. Mol Ther Nucleic Acids.

[25] Liu N, Zhang Z, Li L, Shen X, Sun B, Wang R (2020). MicroRNA-181 regulates the development of Ossification of Posterior longitudinal ligament via Epigenetic Modulation by targeting PBX1. Theranostics.

[26] Xiang J, Fu HQ, Xu Z, Fan WJ, Liu F, Chen B (2020). lncRNA SNHG1 attenuates osteogenic differentiation via the miR101/DKK1 axis in bone marrow mesenchymal stem cells. Mol Med Rep.

[27] Yang S, Guo S, Tong S, Sun X Exosomal miR-130a-3p regulates osteogenic differentiation of Human Adipose-Derived stem cells through mediating SIRT7/Wnt/beta-catenin axis. Cell Prolif. 2020: e12890.

[28] Yin R, Jiang J, Deng H, Wang Z, Gu R, Wang F (2020). miR-140-3p aggregates osteoporosis by targeting PTEN and activating PTEN/PI3K/AKT signaling pathway. Hum Cell.

[29] Zhang L, Li G, Wang K, Wang Y, Dong J, Wang H (2020). MiR-30 family members inhibit osteoblast differentiation by suppressing Runx2 under unloading conditions in MC3T3-E1 cells. Biochem Biophys Res Commun.

[30] Wang Y, Wang K, Hu Z, Zhou H, Zhang L, Wang H (2018). MicroRNA-139-3p regulates osteoblast differentiation and apoptosis by targeting ELK1 and interacting with long noncoding RNA ODSM. Cell Death Dis.

[31] Wang H, Hu Z, Shi F, Dong J, Dang L, Wang Y (2018). Osteoblast-targeted delivery of miR-33-5p attenuates osteopenia development induced by mechanical unloading in mice. Cell Death Dis.

[32] Cao Y, Lv Q, Lv C (2015). MicroRNA-153 suppresses the osteogenic differentiation of human mesenchymal stem cells by targeting bone morphogenetic protein receptor type II. Int J Mol Med.

[33] Bellavia D, De Luca A, Carina V, Costa V, Raimondi L, Salamanna F (2019). Deregulated miRNAs in bone health: Epigenetic roles in osteoporosis. Bone.

[34] Costa V, Carina V, Conigliaro A, Raimondi L, De Luca A, Bellavia D (2019). miR-31-5p Is a LIPUS-Mechanosensitive MicroRNA that Targets HIF-1alpha Signaling and Cytoskeletal Proteins. Int J Mol Sci.

[35] Costa V, Carina V, Raimondi L, De Luca A, Bellavia D, Conigliaro A (2019). MiR-33a Controls hMSCS Osteoblast Commitment Modulating the Yap/Taz Expression Through EGFR Signaling Regulation. Cells.

[36] Hu J, Liao H, Ma Z, Chen H, Huang Z, Zhang Y (2016). Focal Adhesion Kinase Signaling Mediated the Enhancement of Osteogenesis of Human Mesenchymal Stem Cells Induced by Extracorporeal Shockwave. Sci Rep.

[37] Wu J, Zhao J, Sun L, Pan Y, Wang H, Zhang WB (2018). Long non-coding RNA H19 mediates mechanical tension-induced osteogenesis of bone marrow mesenchymal stem cells via FAK by sponging miR-138. Bone.

[38] Eskildsen T, Taipaleenmaki H, Stenvang J, Abdallah BM, Ditzel N, Nossent AY (2011). MicroRNA-138 regulates osteogenic differentiation of human stromal (mesenchymal) stem cells *in vivo*. Proc Natl Acad Sci U S A.

[39] Sun T, Leung F, Lu WW (2016). MiR-9-5p, miR-675-5p and miR-138-5p Damages the Strontium and LRP5-Mediated Skeletal Cell Proliferation, Differentiation, and Adhesion. Int J Mol Sci.

[40] Zhu Z, Tang J, Wang J, Duan G, Zhou L, Zhou X (2016). MiR-138 Acts as a Tumor Suppressor by Targeting EZH2 and Enhances Cisplatin-Induced Apoptosis in Osteosarcoma Cells. PLoS One.

[41] Yu D, An F, He X, Cao X (2015). Curcumin inhibits the proliferation and invasion of human osteosarcoma cell line MG-63 by regulating miR-138. Int J Clin Exp Pathol.

[42] Jiang B, Mu W, Wang J, Lu J, Jiang S, Li L (2016). MicroRNA-138 functions as a tumor suppressor in osteosarcoma by targeting differentiated embryonic chondrocyte gene 2. J Exp Clin Cancer Res.

[43] Tsukamoto S, Lovendorf MB, Park J, Salem KZ, Reagan MR, Manier S (2018). Inhibition of microRNA-138 enhances bone formation in multiple myeloma bone marrow niche. Leukemia.

[44] Li B, Bai L, Shen P, Sun Y, Chen Z, Wen Y (2017). Identification of differentially expressed microRNAs in knee anterior cruciate ligament tissues surgically removed from patients with osteoarthritis. Int J Mol Med.

[45] Zhang L, Yang M, Mayer T, Johnstone B, Les C, Frisch N (2017). Use of MicroRNA biomarkers to distinguish enchondroma from low-grade chondrosarcoma. Connect Tissue Res.

[46] Qu B, Xia X, Wu HH, Tu CQ, Pan XM (2014). PDGF-regulated miRNA-138 inhibits the osteogenic differentiation of mesenchymal stem cells. Biochem Biophys Res Commun.

[47] Feng L, Shi L, Lu YF, Wang B, Tang T, Fu WM (2018). Linc-ROR Promotes Osteogenic Differentiation of Mesenchymal Stem Cells by Functioning as a Competing Endogenous RNA for miR-138 and miR-145. Mol Ther Nucleic Acids.

[48] Li D, Yu K, Xiao T, Dai Y, Liu L, Li H (2019). LOC103691336/miR-138-5p/BMPR2 axis modulates Mg-mediated osteogenic differentiation in rat femoral fracture model and rat primary bone marrow stromal cells. J Cell Physiol.

[49] Hu L, Su P, Yin C, Zhang Y, Li R, Yan K (2018). Microtubule actin crosslinking factor 1 promotes osteoblast differentiation by promoting beta-catenin/TCF1/Runx2 signaling axis. J Cell Physiol.

[50] Zhang Y, Yin C, Hu L, Chen Z, Zhao F, Li D (2018). MACF1 Overexpression by Transfecting the 21 kbp Large Plasmid PEGFP-C1A-ACF7 Promotes Osteoblast Differentiation and Bone Formation. Hum Gene Ther.

[51] Qiu WX, Ma XL, Lin X, Zhao F, Li DJ, Chen ZH (2020). Deficiency of Macf1 in osterix expressing cells decreases bone formation by Bmp2/Smad/Runx2 pathway. J Cell Mol Med.

[52] Zhao F, Ma X, Qiu W, Wang P, Zhang R, Chen Z (2020). Mesenchymal MACF1 Facilitates SMAD7 Nuclear Translocation to Drive Bone Formation. Cells.

[53] Yin C, Zhang Y, Hu L, Tian Y, Chen Z, Li D (2018). Mechanical unloading reduces microtubule actin crosslinking factor 1 expression to inhibit beta-catenin signaling and osteoblast proliferation. J Cell Physiol.

[54] Fassett JT, Xu X, Kwak D, Wang H, Liu X, Hu X (2013). Microtubule Actin Cross-linking Factor 1 regulates cardiomyocyte microtubule distribution and adaptation to hemodynamic overload. PLoS One.

[55] Qian AR, Hu LF, Gao X, Zhang W, Di SM, Tian ZC (2009). Large gradient high magnetic field affects the association of MACF1 with actin and microtubule cytoskeleton. Bioelectromagnetics.

[56] Pagnotti GM, Styner M, Uzer G, Patel VS, Wright LE, Ness KK (2019). Combating osteoporosis and obesity with exercise: leveraging cell mechanosensitivity. Nat Rev Endocrinol.

[57] Mohan S, Wergedal JE, Das S, Kesavan C (2015). Conditional disruption of miR17-92 cluster in collagen type I-producing osteoblasts results in reduced periosteal bone formation and bone anabolic response to exercise. Physiol Genomics.

[58] Chen N, Sui BD, Hu CH, Cao J, Zheng CX, Hou R (2016). microRNA-21 Contributes to Orthodontic Tooth Movement. J Dent Res.

[59] Eimori K, Endo N, Uchiyama S, Takahashi Y, Kawashima H, Watanabe K (2016). Disrupted Bone Metabolism in Long-Term Bedridden Patients. PLoS One.

[60] Holguin N, Brodt MD, Silva MJ (2016). Activation of Wnt Signaling by Mechanical Loading Is Impaired in the Bone of Old Mice. J Bone Miner Res.

