# Supplementary material for: Silencing of miR-138-5p sensitizes bone anabolic action to mechanical stimuli
Source: Theranostics. 2020 Oct 30;10(26):12263–78. doi: 10.7150/thno.53009 (PMC7667683; doi:10.7150/thno.53009)
Supplement: Supplementary file 1 — Supplementary figures and tables. [file thnov10p12263s1.pdf]

SUPPLEMENTARY FIGURES

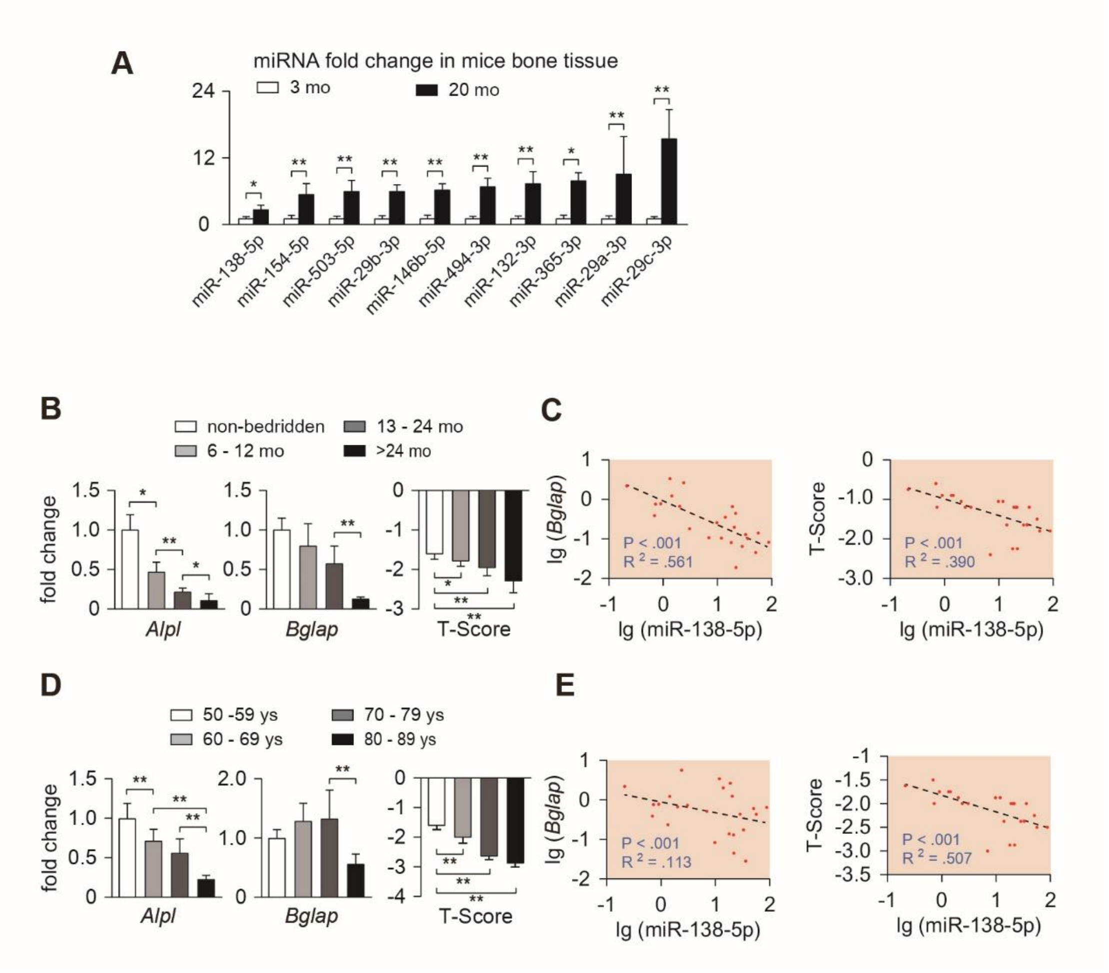

S-Figure 1. Changes of osteogenic marker genes and T-score in bedridden and aged osteoporotic patients. (**A**) Real-time PCR analysis of candidate mechanosensitive miRNAs levels in bone tissues of 3-month-old and 20-month-old mice. n = 4 in each group. (**B**) Real-time PCR analysis of *Alpl* (left) and *Bglap* (middle) levels in bone specimens and T-score (right) of femoral neck from bedridden men patients (50-59 years old) with different periods of immobilization. n = 6 in each group. (**C**) Linear regression analysis of miR-138-5p and *Bglap* (left) and T-score (right) association in bone specimens from bedridden men patients (50-59 years old) with different periods of immobilization. (**D**) Real-time PCR analysis of *Alpl* (left) and *Bglap* (middle) levels in bone specimens and T-score (right) of femoral neck from aged men patients (non-bedridden). n = 6 in each group. (**E**) Linear regression analysis of miR-138-5p and *Bglap* (left) and T-score (right) association in bone specimens from aged men patients (non-bedridden). *Gapdh* was used as the internal control for mRNAs. Data are represented as mean ± sem. Significances were determined using student's *t*-test between two groups. *P* value less than 0.05 were considered significant in all cases (*$P < 0.05$, **$P < 0.01$).

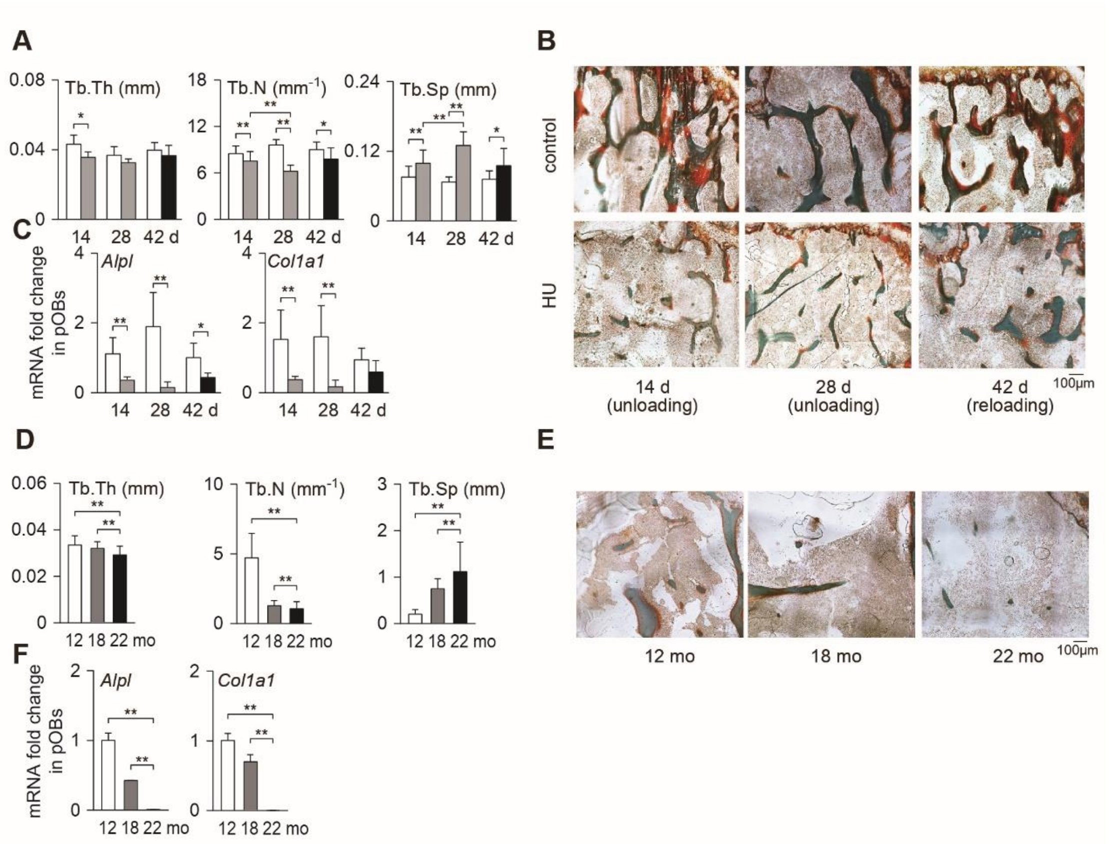

S-Figure 2. Analysis of bone formation in reloaded mice and aged mice. (**A**) MicroCT statistical analysis of trabecular thickness (Tb.Th), trabecular number (Tb.N), trabecular spacing (Tb.Sp), in distal femur of hindlimb unloaded or reloaded mice. n ≥ 6 for each group. (**B**) Representative images of osteoid formation indicated by Goldner's trichrome staining in distal femur of hindlimb unloaded or reloaded mice. Scale bar, 100 µm. (**C**) Real-time PCR analysis of *Alpl* and *Col1a1* mRNA levels in primary osteoblasts isolated from hindlimb unloaded and reloaded mice, respectively. n = 6 for each group. (**D**) MicroCT statistical analysis of trabecular thickness (Tb.Th), trabecular number (Tb.N), trabecular spacing (Tb.Sp), in distal femur of 12, 18 and 22-month-old male C57BL/6 mice. n = 6 for each group. (**E**) Representative images of osteoid formation indicated by Goldner's trichrome staining in distal femur of 12, 18 and 22-month-old male C57BL/6 mice. Scale bar, 100 µm. (**F**) Real-time PCR analysis of *Alpl* and *Col1a1* mRNA levels in primary osteoblasts from 12, 18 and 22-month-old male C57BL/6 mice, respectively. n = 3 for each group. *Gapdh* was used as the internal control for mRNAs. Data are represented as mean ± s.d. Significances were determined using student's *t*-test between two groups. *P* value less than 0.05 was considered significant in all cases (**P* < 0.05, ***P* < 0.01).

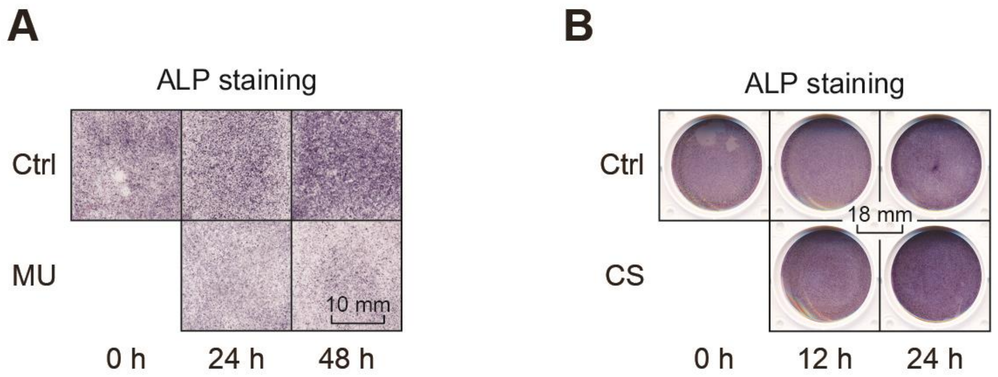

S-Figure 3. Time-course changes of ALP activity in primary osteoblasts under mechanical unloading or cyclic stretch. (**A**) Representative images of ALP staining in primary osteoblasts cultured for 0, 24, and 48 h under mechanical unloading (MU), respectively. Scale bar, 10 mm. (**B**) Representative images of ALP staining in primary osteoblasts cultured for 0, 12, and 24 h under cyclic stretch (CS), respectively. Scale bar, 18 mm.

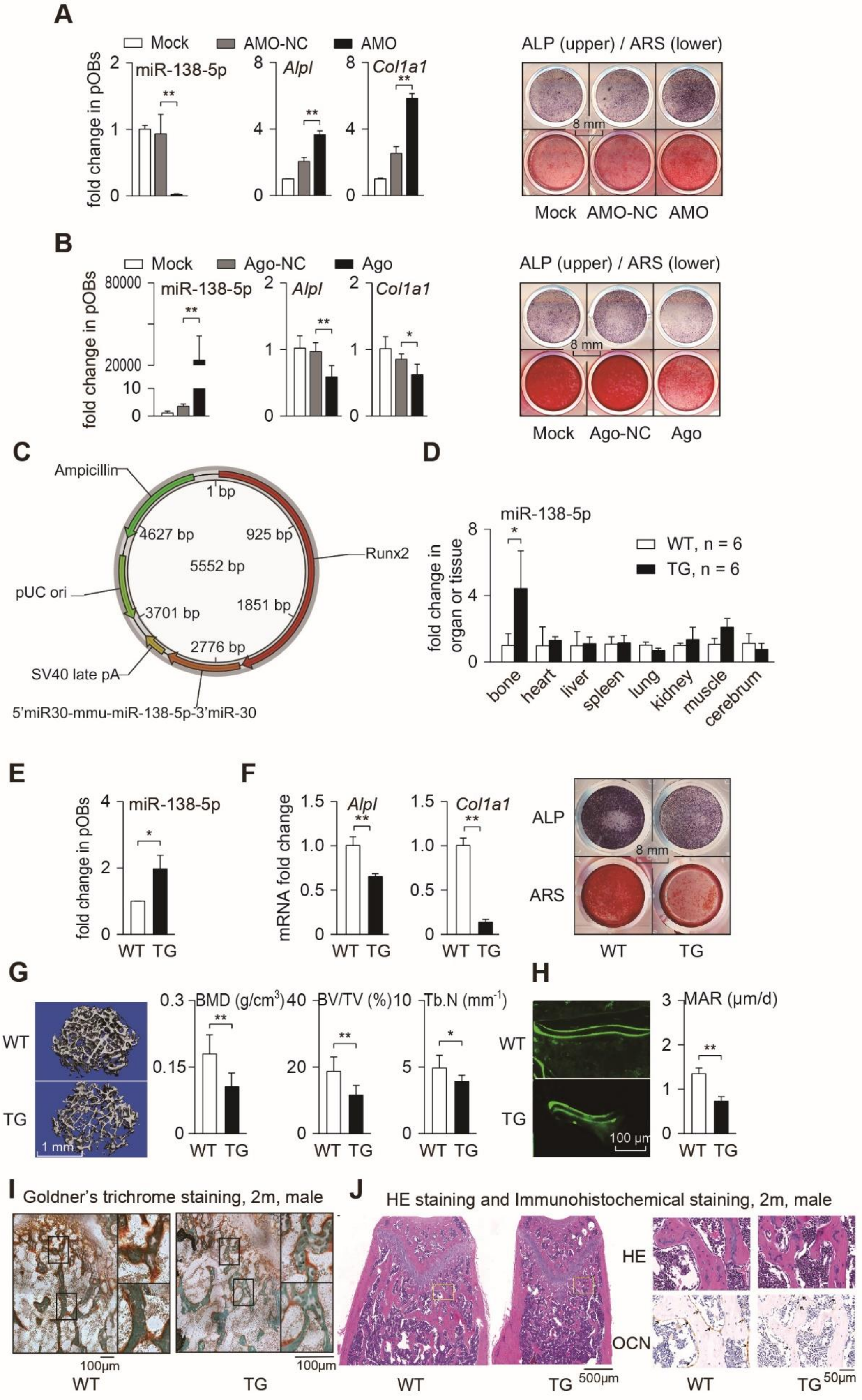
A
Mock
AMO-NC
AMO
fold change in pOBs
miR-138-5p
Alpl
Col1a1
ALP (upper) / ARS (lower)
8 mm
Mock AMO-NC AMO
B
Mock
Ago-NC
Ago
fold change in pOBs
miR-138-5p
Alpl
Col1a1
ALP (upper) / ARS (lower)
8 mm
Mock Ago-NC Ago
C
Ampicillin
1 bp
925 bp
Runx2
4627 bp
5552 bp
pUC ori
1851 bp
3701 bp
2776 bp
SV40 late pA
5'miR30-mmu-miR-138-5p-3'miR-30
D
miR-138-5p
fold change in organ or tissue
WT, n = 6
TG, n = 6
bone
heart
liver
spleen
lung
kidney
muscle
cerebrum
E
miR-138-5p
fold change in pOBs
WT TG
F
mRNA fold change
Alpl
Col1a1
ALP
ARS
8 mm
WT TG
G
WT
TG
1 mm
BMD (g/cm3)
BV/TV (%)
Tb.N (mm-1)
WT TG
H
WT
TG
100 μm
MAR (μm/d)
WT TG
I
Goldner's trichrome staining, 2m, male
100μm
WT
TG
J
HE staining and Immunohistochemical staining, 2m, male
HE
OCN
WT
TG
500μm
50μm

S-Figure 4. miR-138-5p negatively regulates osteoblast differentiation and bone formation. (**A**) Real-time PCR analysis of miR-138-5p (left) and osteogenic marker genes *Alpl* and *ColIa1* mRNA levels (middle), and representative images of ALP staining (right, upper) and Alizarin Red S (ARS) staining (right, lower, 10 d) in primary osteoblasts treated with miR-138-5p antagonist (AMO). Scale bar, 8 mm. (**B**) Real-time PCR analysis of miR-138-5p (left) and osteogenic marker genes *Alpl* and *ColIa1* mRNA levels (middle), and representative images of ALP staining (right, upper) and ARS staining (right, lower, 14 d) in primary osteoblasts treated with miR-138-5p agonist (Ago). Scale bar, 8 mm. (**C**) The Recombinant (pRP (Exp)-RUNX2>5'miR30-mmu-miR-138-5p-3'miR30) plasmid was constructed with the Runx2 promotor. (**D**) Real-time PCR analysis of miR-138-5p level in organs or tissues from 2-month male wild type (WT) and osteoblastic miR-138-5p transgenic (TG) mice. (**E**) Real-time PCR analysis of miR-138-5p level in primary osteoblasts isolated from 2-month male WT and TG mice. (**F**) Real-time PCR analysis of *Alpl* and *ColIa1* mRNA levels (left), and representative images of ALP staining (right, upper) and ARS staining (right, lower, 14 d) in primary osteoblasts isolated from 2-month male WT and TG mice. Scale bar, 8 mm. (**G**) Representative 3D reconstruction images showing microarchitecture (left), and microCT statistical analysis of BMD, BV/TV, Tb.N (right) in distal femur of 2-month male WT and TG mice. BMD, bone mineral density; BV/TV, bone volume fraction; Tb.N, trabecular number. Scale bar, 1 mm. (**H**) Representative calcein double labeling images (left) and dynamic histomorphometric analysis of MAR (right) showing bone formation capacity in distal femur of 2-month male WT and TG mice. Scale bar, 100 μm. (**I**) Representative images of osteoid formation indicated by Goldner's trichrome staining in distal femur of 2-month male WT and TG mice. Scale bar, 100 μm. (**J**) Representative images of H&E staining and immunohistochemical staining analysis of osteocalcin (OCN) in distal femur of 2-month male WT and TG mice. Scale bar, 500μm and 50 μm. U6 small nuclear RNA was used as the internal control for miR-138-5p, and *Gapdh* was used as the internal control for mRNAs. For mice, $n \geq 6$ in each group. For cell experiments, $n = 3$ in each group for Real-time PCR analysis. Data are represented as mean ± s.d. Significances were determined using student's *t*-test between two groups. *P* value less than 0.05 was considered significant in all cases (*$P < 0.05$, **$P < 0.01$).

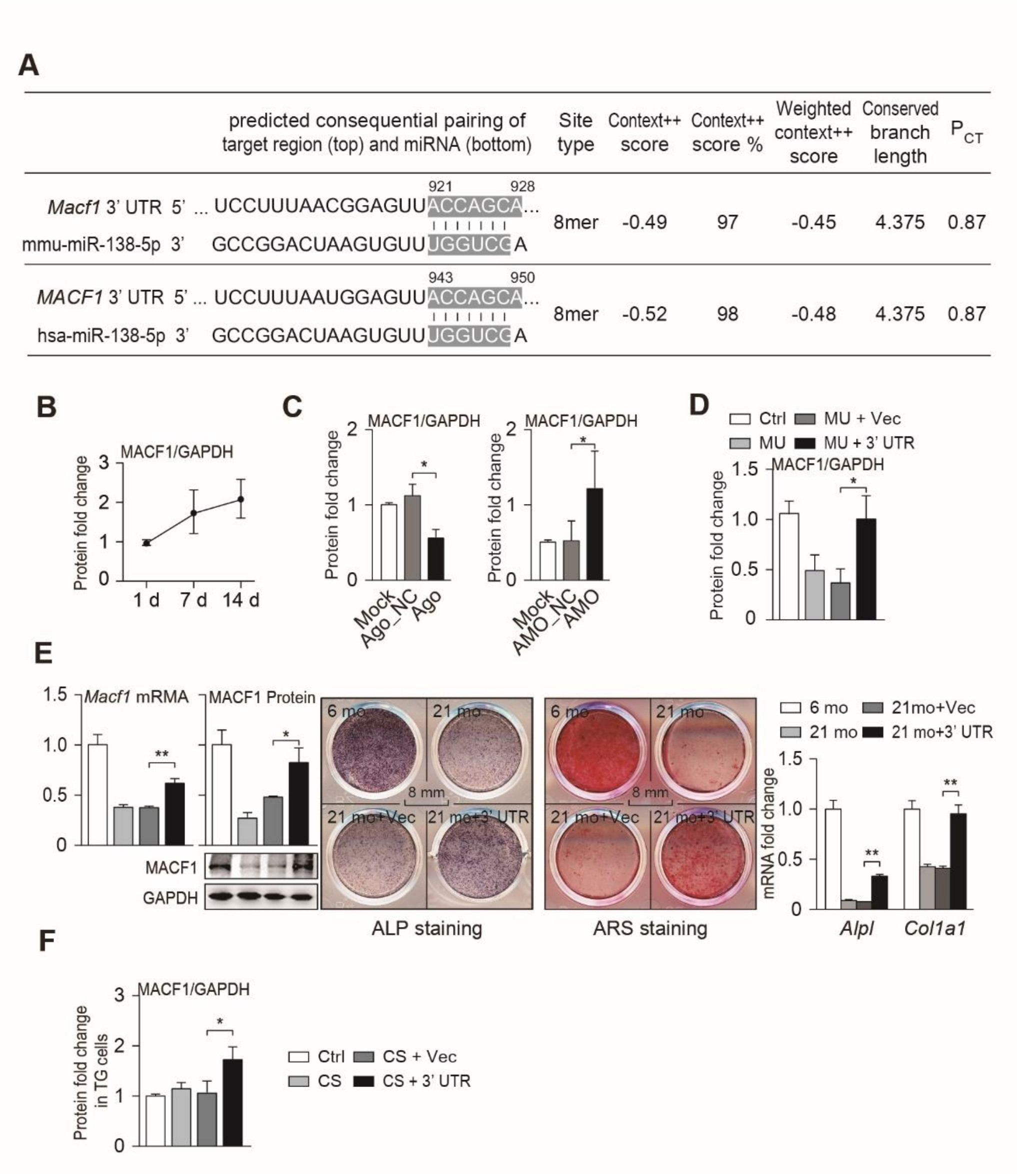

S-Figure 5. miR-138-5p targets MACF1 to inhibit aged osteoblast differentiation. (**A**) Sequence alignments between miR-138-5p and candidate binding sites in the *Macf1* 3' UTR predicted with the TargetScan database. (**B**) Quantification of MACF1 protein level in primary osteoblasts during osteo-induction. (**C**) Quantification of MACF1 protein level in primary osteoblasts treated with either miR-138-5p agonist (Ago) or antagonist (AMO), respectively. (**D**) Quantification of MACF1 protein level in primary osteoblasts treated with *Macf1* 3'UTR plasmid or blank vector under MU condition, respectively. (**E**) Real-time PCR and western blot (left) analysis of MACF1 levels, representative images of ALP staining (middle, left) and ARS staining (middle, right, 16 d), and Real-time PCR analysis of *Alpl* and *ColIa1* mRNA levels (right) in primary 21-month aged osteoblasts treated with *Macf1* 3'UTR plasmid or blank vector. Scale bar, 8 mm. (**F**) Quantification of MACF1 level in TG cells treated with *Macf1* 3' UTR plasmid or blank vector under CS condition, respectively. *Gapdh* was used as the internal control for mRNAs. n = 3 in each group for Real-time PCR and Western Blot analysis. Data are represented as mean ± s.d. Significances were determined using student's *t*-test between two groups. *P* value less than 0.05 were considered significant in all cases (*$P < 0.05$, **$P < 0.01$).

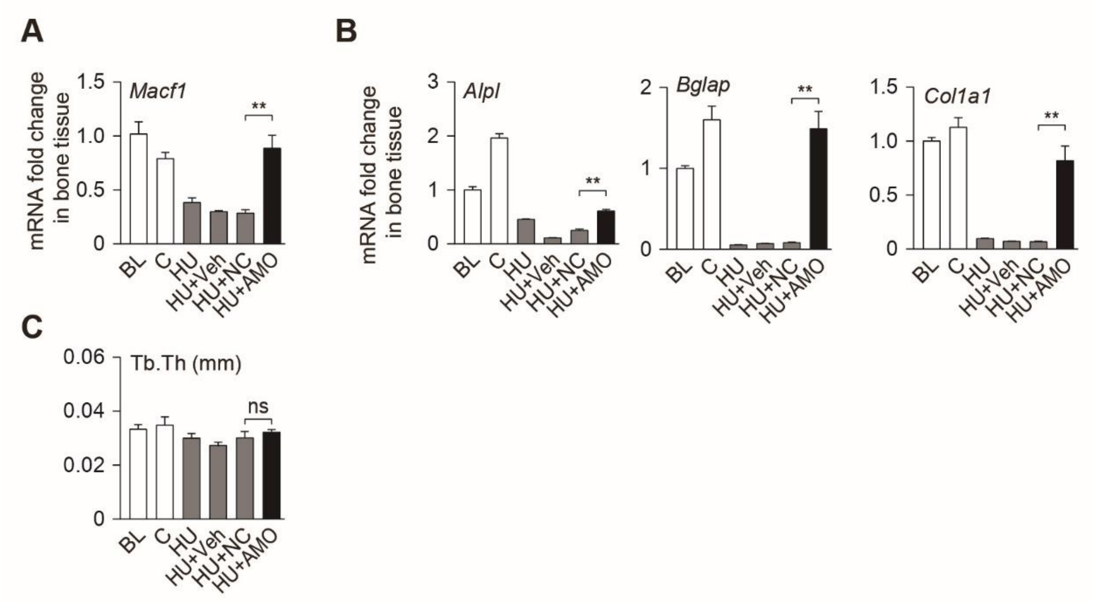

S-Figure 6. miR-138-5p antagonist restores unloading-induced decrease of bone formation. (**A**) Real-time PCR analysis of *Macf1* mRNA levels in bone tissue of all groups. n = 4 in each group. BL (baseline, 3-month-old mice), C (4-month-old mice, without any injection), HU (4-month-old mice, hindlimb unloading for 28d without any injection), HU + Veh (4-month-old mice, hindlimb unloading for 28d and injected with osteoblast-targeted delivery system), HU + NC (4-month-old mice, hindlimb unloading for 28d and injected with both osteoblast-targeted delivery system and antagomir-NC), HU + AMO (4-month-old mice, hindlimb unloading for 28d and injected with both osteoblast-targeted delivery system and antagomir-138-5p). (**B**) Real-time PCR analysis of *Alpl*, *Col1a1* and *Bglap* mRNA levels in bone tissue of all groups. n = 4 in each group. (**C**) microCT statistical analysis of trabecular thickness (Tb.Th) in distal femur of hindlimb unloaded mice pretreated with AMO. n ≥ 5 mice for each group. *Gapah* was used as the internal control for mRNA. Data are represented as mean ± s.d. Significances were determined using student's *t*-test between two groups. *P* value less than 0.05 was considered significant in all cases (***P* < 0.01).

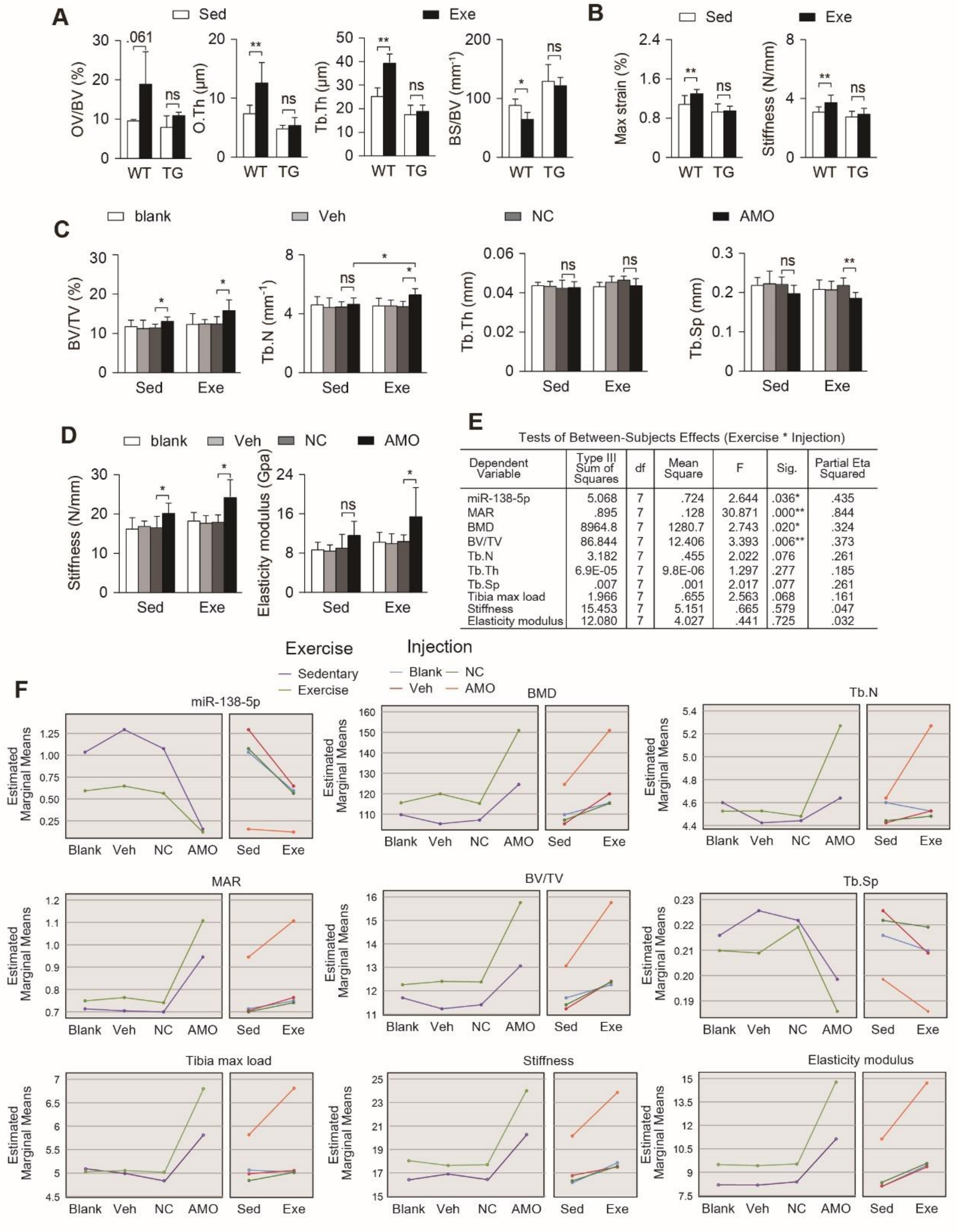

Tests of Between-Subjects Effects (Exercise * Injection)

Dependent Variable	Type III Sum of Squares	df	Mean Square	F	Sig.	Partial Eta Squared
miR-138-5p	5.068	7	.724	2.644	.036*	.435
MAR	.895	7	.128	30.871	.000**	.844
BMD	8964.8	7	1280.7	2.743	.020*	.324
BV/TV	86.844	7	12.406	3.393	.006**	.373
Tb.N	3.182	7	.455	2.022	.076	.261
Tb.Th	6.9E-05	7	9.8E-06	1.297	.277	.185
Tb.Sp	.007	7	.001	2.017	.077	.261
Tibia max load	1.966	7	.655	2.563	.068	.161
Stiffness	15.453	7	5.151	.665	.579	.047
Elasticity modulus	12.080	7	4.027	.441	.725	.032

S-Figure 7. miR-138-5p overexpression loses mechano-sensitization of anabolic bone action in miR-138-5p transgenic osteoporotic mice. (**A**) Statistical analysis of osteoid parameters (OV/BV and O.Th) and trabecular parameters (Tb.Th and BS/BV) in distal femur of miR-138-5p transgenic (TG) mice and wild type (WT) mice after 4-week treadmill exercise. Sed, sedentary, Exe, exercise. n = 4 mice for each group. OV/BV: osteoid volume per bone volume; O.Th: osteoid thickness; Tb.Th: trabecular thickness; BS/BV: Bone surface per bone volume. (**B**) Three-point bending measurement of femur maximum strain and stiffness in femur of WT and TG mice after treadmill exercise. n ≥ 5 mice for each group. (**C**) MicroCT analysis of BV/TV, Tb.N, Tb.Th, Tb.Sp in distal femur after treadmill exercise and AMO treatment. n = 6 for each group. BV/TV, bone volume fraction; Tb.N, trabecular number; Tb.Th, trabecular thickness; Tb.Sp, trabecular spacing. Blank (without any injection), Veh (injected with osteoblast-targeted delivery system), NC (injected with both osteoblast-targeted delivery system and antagomir-NC), AMO (injected with both osteoblast-targeted delivery system and antagomir-138-5p). Sed, sedentary, Exe, exercise. (**D**) Three-point bending measurement of tibia stiffness and elasticity modulus in femur after treadmill exercise and AMO treatment. n = 6 for each group. Data are represented as mean ± s.d. Significances were determined using student's *t*-test between two groups. *P* value less than 0.05 was considered significant in all cases (ns, no difference, **P* < 0.05, ***P* < 0.01). (**E**) Tests of Between-subjects Effects (Exercise and injection) of miR-138-5p, MAR, BMD, BV/TV, Tb.N, Tb.Th, Tb.Sp, tibia max load, stiffness and elasticity modulus in all groups. (**F**) Two-way ANOVA analysis of Estimated Marginal Means of miR-138-5p, MAR, BMD, BV/TV, Tb.N, Tb.Sp, tibia max load, stiffness and elasticity modulus in all groups.

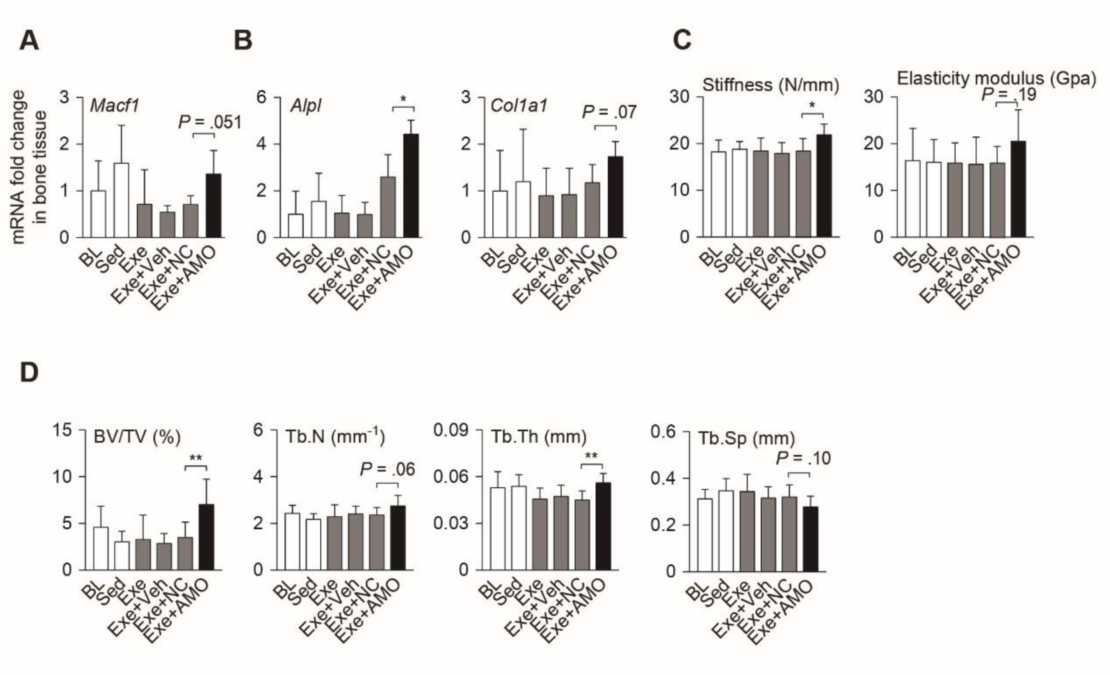

S-Figure 8. miR-138-5p antagonist sensitizes anabolic bone action to mechanical stimuli in aged osteoporotic mice. (**A**) Real-time PCR analysis of *Macf1* mRNA level in bone tissue of all groups. n = 4 in each group. BL (baseline, 20-month-old mice), Sed (21-month-old mice, without any injection and without treadmill), Exe (21-month-old mice, treatment with treadmill exercise and without any injection), Exe + Veh (21-month-old mice, treatment with treadmill exercise and injected with osteoblast-targeted delivery system), Exe + NC (21-month-old mice, treatment with treadmill exercise and injected with osteoblast-targeted delivery system and antagomir-NC), Exe + AMO (21-month-old mice, treatment with treadmill exercise and injected with osteoblast-targeted delivery system and antagomir-138-5p). (**B**) Real-time PCR analysis of *Alpl* and *Col1a1* mRNA levels in bone tissue of all groups. n = 4 in each group. (**C**) Three-point bending measurement of tibia stiffness and elasticity modulus in femur of of all groups. n ≥ 5 mice for each group. (**D**) MicroCT analysis of BV/TV, Tb.N, Tb.Th, Tb.Sp in distal femur after treadmill exercise and AMO treatment. n ≥ 5 mice for each group. BV/TV, bone volume fraction; Tb.N, trabecular number; Tb.Th, trabecular thickness; Tb.Sp, trabecular spacing. *Gapah* was used as the internal control for mRNA. Data are represented as mean ± s.d. Significances were determined using student's *t*-test between two groups. *P* value less than 0.05 was considered significant in all cases (*$P < 0.05$, **$P < 0.01$).

S-Table 1 Clinical features of patients involved in bone specimens analysis

Patient	Age	Gender	Bedridden time (months)	*T*-score for femoral neck
1	55	Male	NA	-1.6
2	57	Male	NA	-1.8
3	54	Male	NA	-1.5
4	52	Male	NA	-1.7
5	58	Male	NA	-1.4
6	59	Male	NA	-1.6
7	54	Male	6	-1.8
8	57	Male	8	-1.8
9	55	Male	9	-1.7
10	51	Male	7	-1.6
11	59	Male	10	-1.8
12	54	Male	11	-2
13	53	Male	14	-2.2
14	55	Male	16	-1.8
15	56	Male	15	-1.7
16	57	Male	18	-1.8
17	54	Male	20	-2.1
18	58	Male	21	-2.1
19	53	Male	25	-1.8
20	52	Male	26	-2.5
21	54	Male	26	-2.2
22	56	Male	27	-2.6
23	56	Male	25	-2.1
24	57	Male	28	-2.5
25	67	Male	NA	-1.9
26	63	Male	NA	-2.1
27	62	Male	NA	-1.8
28	68	Male	NA	-2.3
29	66	Male	NA	-2.8
30	68	Male	NA	-2.1
31	76	Male	NA	-2.6
32	72	Male	NA	-2.5
33	77	Male	NA	-2.7
34	76	Male	NA	-2.5
35	76	Male	NA	-2.7
36	78	Male	NA	-2.8
37	88	Male	NA	-3.1
38	86	Male	NA	-2.9
39	84	Male	NA	-2.8
40	83	Male	NA	-2.7
41	84	Male	NA	-2.8
42	88	Male	NA	-2.9
